# Correction: Small is beautiful, but large is certified: A comparison between fisheries the Marine Stewardship Council (MSC) features in its promotional materials and MSC-certified fisheries

**DOI:** 10.1371/journal.pone.0253486

**Published:** 2021-06-14

**Authors:** Frédéric Le Manach, Jennifer L. Jacquet, Megan Bailey, Charlène Jouanneau, Claire Nouvian and the PLOS ONE Editors

After this article [1] was published, the Marine Stewardship Council (MSC) raised concerns that the article’s competing interests and funding statements are not complete, and that the article includes claims that are not supported by the reported data or published literature. The competing interest and funding concerns involved transparency about interests involving BLOOM, a non-profit organization that has publicly criticized the MSC and whose activities include assessment of seafood labels and certification programs (including those of the MSC).

PLOS followed up on the concerns and conducted a post-publication editorial assessment to evaluate the potential impacts of undisclosed competing interests on the article’s peer review. The outcome of this assessment supported the conclusion that the disclosure issues did not unduly impact the peer review process; a member of *PLOS ONE*’s Editorial Board advised in the post-publication discussions that they did not have concerns about the study design or analyses. However, *PLOS ONE* concluded that the following updates and clarifications are needed.

FLM and CN listed their affiliations with BLOOM in the article’s “About the Authors” section but the published Competing Interest statement says, “The authors have declared that no competing interests exist.” After careful review, the *PLOS ONE* Editors concluded that based on available information about BLOOM’s activities, the affiliations of FLM and CN and other previously undisclosed associations to BLOOM and the MSC need to be declared as potential competing interests. Accordingly, the article’s Competing Interests statement is updated to:

As noted in the article’s “About the Authors” section, FLM and CN are affiliated with BLOOM. CN is Founder and Honorary President of BLOOM. As such, she has never received any salary from BLOOM from its inception in 2005 to 2020. FLM is the organization’s Scientific Director. CJ worked for BLOOM from 2014–2016, and JJ was an unpaid member of BLOOM’s Board of Directors from 2010–2012. FLM was a member of the public chamber of the MSC Stakeholder Council in 2016–2017 and received travel funds from the MSC to attend a meeting for this Council.

The Data Availability Statement is updated to:

All data and processing scripts are available from http://dx.doi.org/10.17632/gpynbmn7f9.1. The authors did not obtain specific permissions to publish or make publicly available the primary images, which are available through the Mendeley repository. These images are all screenshots from public reports and social media, and are not covered by the *PLOS ONE* article’s CC-BY 4.0 license.

Sentences 3 and 4 in the Abstract are revised to cite sources and identify assertions as hypotheses:

**Original:** “While the MSC is increasingly recognized by decision-makers as an indicator for fishery success, it is also criticized for weak standards and overly-lenient third-party certifiers. This gap between the standard’s reputation and its actual implementation could be a result of how the MSC markets and promotes its brand.”
**Revised version:** “While the MSC is increasingly recognized by decision-makers as an indicator for fishery success, it has also been criticized for having weak standards and lenient third-party certifiers (e.g. see [7, 22]). We hypothesized that these disparate views of the MSC’s reputation and program implementation could be a result of how the MSC markets and promotes its brand.”

In the Introduction, the study aim statement is revised to clearly and accurately describe the reported study.

**Original:** “As with agricultural production, there is a widespread perception that ‘small is beautiful’ [37–40], which led us to question whether the MSC was accurately representing its certified fisheries in its promotional materials.”
**Revised version:** “As with agricultural production, there is a widespread perception among consumers that ‘small is beautiful’ [37–40]. In this study, we assessed images used in MSC promotional materials and compared the relative proportion of different fishery types shown in these materials to the relative abundance of different fishery types certified by the MSC.”

The following paragraphs are added to the article’s Discussion to address some of the study’s limitations:

The study analyzed images only, it did not assess the materials’ overall communications, including text discussion, about different types of fisheries, their representation in the overall portfolio of MSC-certified fisheries.
Conclusions cannot be drawn from this study’s results as to the MSC’s objectives in selecting images to show in their materials, or about why there are differences between the relative abundance of different fishery types shown in marketing materials versus certified by the MSC. Images used in promotional materials may be selected for various reasons, such as the company’s marketing objectives, the intended audience of the materials, and/or to represent aspects of the message(s) relayed in accompanying text.

Readers should note that the following text in the Discussion represents the authors’ opinions and is not intended to relay conclusions following from the data reported in the article.

“…it appears that the MSC strongly appeals to the idealization of fisheries by consumers and policy-makers by promoting fisheries involving small-scale gears and passive gears in much higher proportions than in reality, as is the case in other sectors such as agriculture [37–39]. The MSC may be trying to appeal to the needs and desires of its consumers, providing them with the symbolic satisfaction of not having harmed the environment [58].”
“there is a credible risk of misunderstanding for those who scroll quickly through their documents and websites, which may explain to some extent the positive public image of the MSC.”
“We hypothesize that this discrepancy between MSC-certified fisheries and what the MSC advertises aims to ‘green’ its image with consumers. We further posit that this discrepancy might be the reason behind a perceived gap between its ‘supporters’—including policy-makers—and other stakeholders that have gradually disengaged or become critical of the MSC (e.g., coalitions such as On The Hook or Make Stewardship Count).”

*Except for Reference #1, the citations included in this notice refer to the published article’s References list.
