# Supplementary material for: Small is beautiful, but large is certified: A comparison between fisheries the Marine Stewardship Council (MSC) features in its promotional materials and MSC-certified fisheries
Source: PLoS One. 2020 May 4;15(5):e0231073. doi: 10.1371/journal.pone.0231073 (PMC7197776; doi:10.1371/journal.pone.0231073)
Supplement: S1 Text — (DOCX) [file pone.0231073.s001.docx]

**S1 Text. Description of the process used to produce a time-series of the MSC-certified catch.**

Annual catch data for all certified fisheries were collected according to the process described below (visual representation in S1 Figure 1):

1. Reference catches were collected from the MSC's website (<https://fisheries.msc.org/en/fisheries>);
2. When these unique annual reference catches were not available on the MSC's website, the latest available data were extracted from either 'public comment (draft) reports' (PC(D)R) or 'annual surveillance reports' (SR), also available on the same website (see S2 Table 1). Reference catches from the website were similarly updated when they were over three years older than the last year of the fishery's certification (i.e. 2017, if still certified);
3. Fisheries were then sorted by updated annual reference catch. For those accounting for 90% of the total MSC-certified catch (74 fisheries; see S2 Table 1), annual reference catches were replaced by full time-series reconstructed from the reports mentioned in point 2. Note that extrapolations and interpolations were made in two instances (see the 'annual catch data.xlsx' file available in the online repository linked to this article: <http://bit.ly/2K426Nz>):
4. when annual catches could not be found for the earlier or later years, the closest annual catch was carried backward/forward; and
5. when there was a gap in the time-series, the average of the surrounding annual catches was used.

We deemed that the annual variations in the many small fisheries accounting for the remaining 10% of the total MSC-certified catch as of 2017 would not affect the overall trends in a substantial manner. Therefore, annual catches were not collated for these fisheries.

**NB: given that this assumption 'excludes' most small-scale fisheries, the analyses were also conducted in terms of 'number' of fisheries and not only in terms of 'catch' (see main manuscript).**


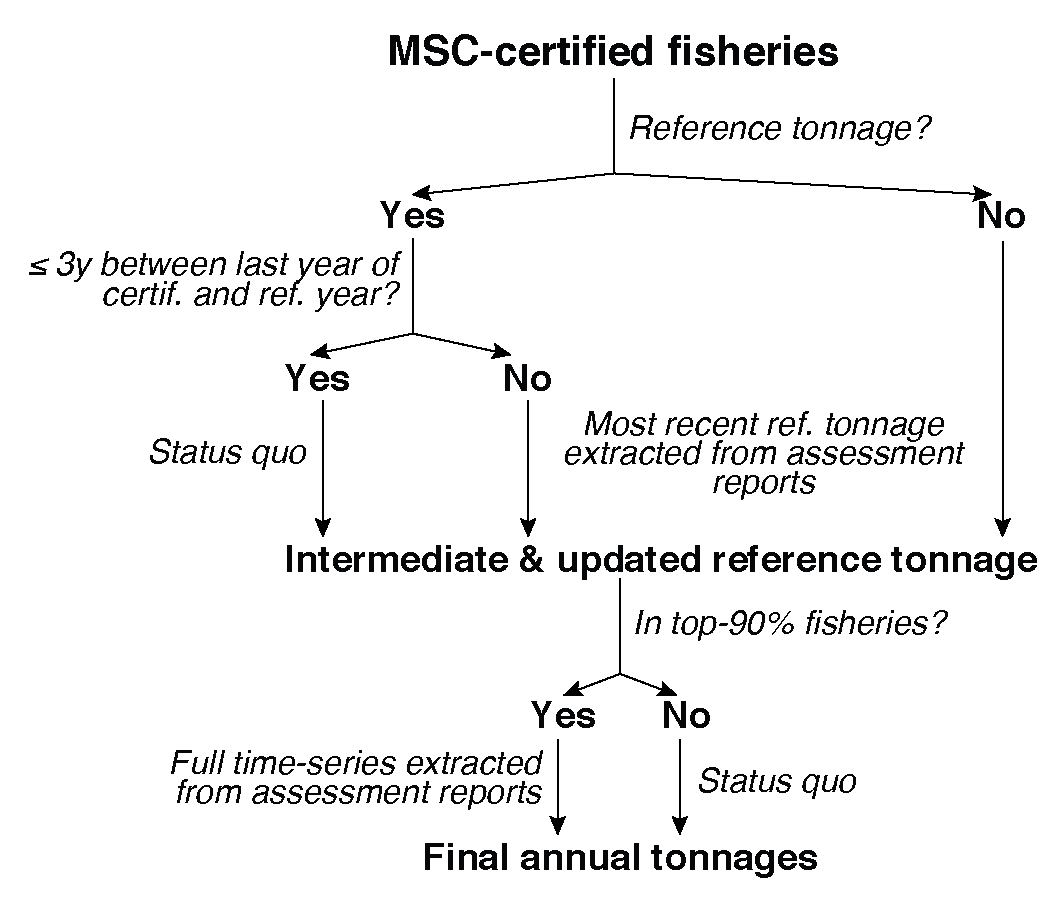


S1 Figure 1. Diagram explaining how annual were estimated, based on the information available on the MSC's website ([www.fisheries.msc.org](http://www.fisheries.msc.org)) as well as in assessment reports (i.e. 'public comment reports' and annual 'surveillance reports').
