# Supplementary material for: Small is beautiful, but large is certified: A comparison between fisheries the Marine Stewardship Council (MSC) features in its promotional materials and MSC-certified fisheries
Source: PLoS One. 2020 May 4;15(5):e0231073. doi: 10.1371/journal.pone.0231073 (PMC7197776; doi:10.1371/journal.pone.0231073)
Supplement: S1 Table — (DOC) [file pone.0231073.s002.doc]

| **S2 Table 1.** Summary of the MSC-certified fisheries, as of 31 December 2017 (ordered by certification date within each status category).   |  | | --- | |  | | | | | | | | | | | | | |
| --- | --- | --- | --- | --- | --- | --- | --- | --- | --- | --- | --- | --- |
| **Status** | **Name of the fishery** | **Comment** | **Certif. date** | **Suspension date** | **Objection** | **Reference catch** | **Reference year** | **Top 90%** | **Source** | | |  |
|  |  |  |  |  |  |  |  |  | **PC(D)R** | **SR** | **Website** |  |
| Certified | Australian Western rock lobster |  | Mar 2000 |  |  | 5 947.0 | 2015 | No |  |  | ✓ |  |
|  | Alaska salmon |  | Oct 2000 |  |  | 294 520.0 | 2016 | Yes |  |  | ✓ |  |
|  | Burry Inlet cockles |  | Apr 2001 |  |  | 996.0 | 2016 | No |  |  | ✓ |  |
|  | New Zealand hoki |  | Nov 2001 |  | Yes | 136 718.0 | 2016 | Yes |  | ✓ |  |  |
|  | South Georgia Patagonian toothfish longline |  | Mar 2004 |  | Yes | 2 192.0 | 2017 | No |  |  | ✓ |  |
|  | South Africa hake trawl |  | Apr 2004 |  |  | 132 207.0 | 2016 | Yes |  | ✓ |  |  |
|  | Mexico Baja California red rock lobster |  | Apr 2004 |  |  | 1 172.2 | 2015 | No |  |  | ✓ |  |
|  | Alaska pollock Bering Sea and Aleutian Islands |  | Feb 2005 |  | Yes | 1 347 947.0 | 2016 | Yes |  | ✓ |  |  |
|  | Alaska pollock Gulf of Alaska |  | Apr 2005 |  | Yes | 176 989.0 | 2016 | Yes |  | ✓ |  |  |
|  | Hastings fleet Dover sole and plaice | Could not find any evidence that this fishery had withdrawn | Sep 2005 |  |  | 97.2 | 2014 | No |  |  | ✓ |  |
|  | Australia mackerel icefish |  | Mar 2006 |  |  | 469.0 | 2016 | No |  |  | ✓ |  |
|  | US North Pacific halibut |  | Apr 2006 |  |  | 8 079.0 | 2014 | No |  |  | ✓ |  |
|  | US North Pacific sablefish |  | May 2006 |  |  | 9 683.0 | 2015 | No |  |  | ✓ |  |
|  | Lake Hjälmaren pikeperch fish-trap and gillnet |  | Aug 2006 |  |  | 201.2 | 2016 | No |  |  | ✓ |  |
|  | Patagonian scallop (*Zygochlamys patagonica*) bottom otter trawl |  | Dec 2006 |  |  | 32 282.1 | 2016 | Yes |  |  | ✓ |  |
|  | Norway North East Arctic saithe |  | Jun 2008 |  |  | 115 775.0 | 2015 | Yes |  |  | ✓ |  |
|  | SPFPO Swedish North Sea herring |  | Jun 2008 |  |  | 16 162.0 | 2016 | No |  |  | ✓ |  |
|  | Canada Scotian Shelf Northern prawn trawl |  | Aug 2008 |  |  | 4 111.0 | 2016 | No |  |  | ✓ |  |
|  | Germany North Sea saithe trawl |  | Oct 2008 |  |  | 7 920.5 | 2016 | No |  |  | ✓ |  |
|  | Norway North Sea and Skagerrak herring |  | Apr 2009 |  |  | 146 350.0 | 2015 | Yes |  |  | ✓ |  |
|  | Ekofish Group-North Sea twin rigged otter trawl plaice |  | Jun 2009 |  |  | 2 361.0 | 2014 | No | ✓ |  |  |  |
|  | DPPO and DFPO North Sea herring |  | Jun 2009 |  |  | 113 260.0 | 2016 | Yes |  |  | ✓ |  |
|  | Iturup Island pink & chum salmon |  | Sep 2009 |  |  | 15 834.0 | 2016 | No |  | ✓ |  |  |
|  | Canada Pacific halibut (British Columbia) |  | Sep 2009 |  |  | 3 713.0 | 2016 | No |  | ✓ |  |  |
|  | Vietnam Ben Tre clam hand gathered |  | Nov 2009 |  |  | 4 341.0 | 2014 | No |  |  | ✓ |  |
|  | Alaska Pacific cod Bering Sea and Aleutian Islands |  | Jan 2010 |  |  | 220 956.0 | 2016 | Yes |  | ✓ |  |  |
|  | Alaska Pacific cod Gulf of Alaska |  | Jan 2010 |  |  | 58 846.0 | 2016 | Yes |  | ✓ |  |  |
|  | Faroese Pelagic Organisation Atlanto-Scandian herring |  | Feb 2010 |  |  | 36 381.0 | 2014 | Yes |  |  | ✓ |  |
|  | Scapeche, Euronor and Compagnie de Peche de St Malo saithe |  | Mar 2010 |  |  | 13 770.2 | 2014 | No |  |  | ✓ |  |
|  | Eastern Canada offshore scallop |  | Mar 2010 |  |  | 35 615.4 | 2016 | Yes |  |  | ✓ |  |
|  | Canada Highly Migratory Species Foundation (CHMSF) British Columbia albacore tuna North Pacific |  | Mar 2010 |  |  | 2 842.0 | 2016 | No |  | ✓ |  |  |
|  | Norway North East Arctic cod |  | Apr 2010 |  |  | 465 164.0 | 2015 | Yes |  |  | ✓ |  |
|  | Norway North East Arctic haddock |  | Apr 2010 |  |  | 91 264.0 | 2015 | Yes |  |  | ✓ |  |
|  | Alaska flatfish Bering Sea and Aleutian Islands |  | May 2010 |  |  | 213 350.0 | 2016 | Yes |  | ✓ |  |  |
|  | Alaska flatfish Gulf of Alaska |  | May 2010 |  |  | 24 178.0 | 2016 | No |  | ✓ |  |  |
|  | Eastern Canada offshore lobster |  | Jun 2010 |  |  | 788.5 | 2016 | No |  | ✓ |  |  |
|  | Aker Biomarine Antarctic krill |  | Jun 2010 |  | Yes | 155 301.0 | 2016 | Yes |  | ✓ |  |  |
|  | Cornwall sardine, UK |  | Jun 2010 |  |  | 2 828.0 | 2014 | No |  |  | ✓ |  |
|  | North West Atlantic Canada harpoon swordfish |  | Jun 2010 |  |  | 110.5 | 2016 | No |  |  | ✓ |  |
|  | South Brittany sardine purse seine |  | Jul 2010 |  |  | 15 887.0 | 2017 | No |  | ✓ |  |  |
|  | South Georgia icefish pelagic trawl |  | Oct 2010 |  |  | 49.1 | 2017 | No |  |  | ✓ |  |
|  | Canada Scotia-Fundy haddock |  | Oct 2010 |  |  | 15 325.1 | 2016 | No |  | ✓ |  |  |
|  | North Menai Strait mussel |  | Oct 2010 |  |  | 4 940.0 | 2016 | No |  |  | ✓ |  |
|  | OCI Grand Bank yellowtail flounder trawl |  | Oct 2010 |  |  | 17 000.0 | 2015 | No |  |  | ✓ |  |
|  | Ross Sea toothfish longline |  | Nov 2010 |  | Yes | 4 172.0 | 2017 | No |  |  | ✓ |  |
|  | Oregon and Washington pink shrimp |  | Nov 2010 |  |  | 23 947.0 | 2017 | No |  |  | ✓ |  |
|  | Pacific hake mid-water trawl |  | Nov 2010 |  | Yes | 329 428.0 | 2016 | Yes |  | ✓ |  |  |
|  | Barents Sea cod, haddock and saithe |  | Nov 2010 |  |  | 135 495.0 | 2016 | Yes |  |  | ✓ |  |
|  | UK Fisheries/DFFU/Doggerbank Group saithe |  | Jan 2011 |  |  | 2 318.9 | 2016 | No |  |  | ✓ |  |
|  | DFPO Denmark North Sea & Skagerrak cod & saithe |  | Feb 2011 |  |  | 7 207.0 | 2015 | No |  |  | ✓ |  |
|  | DFPO Denmark North Sea plaice |  | Mar 2011 |  | Yes | 10 022.7 | 2015 | No |  |  | ✓ |  |
|  | New Zealand albacore tuna troll |  | May 2011 |  | Yes | 2 225.0 | 2015 | No |  |  | ✓ |  |
|  | Normandy and Jersey lobster |  | Jun 2011 |  |  | 437.0 | 2016 | No |  |  | ✓ |  |
|  | Annette Islands Reserve salmon |  | Jun 2011 |  |  | 4 426.6 | 2015 | No |  |  | ✓ |  |
|  | Tristan da Cunha rock lobster |  | Jun 2011 |  |  | 387.0 | 2017 | No |  |  | ✓ |  |
|  | Canada northern and striped shrimp |  | Jun 2011 |  |  | 98 001.0 | 2015 | Yes |  |  | ✓ |  |
|  | Spencer Gulf king prawn |  | Jul 2011 |  |  | 2 180.0 | 2016 | No |  | ✓ |  |  |
|  | Netherlands blue shell mussel |  | Jul 2011 |  |  | 42 294.1 | 2016 | Yes |  |  | ✓ |  |
|  | Argentine anchovy (*Engraulis anchoita*), Bonaerense stock, semi-pelagic mid-water trawl |  | Aug 2011 |  |  | 3 649.4 | 2017 | No |  |  | ✓ |  |
|  | Suriname Atlantic seabob shrimp |  | Nov 2011 |  | Yes | 6 310.0 | 2016 | No |  |  | ✓ |  |
|  | PNA Western and Central Pacific skipjack and yellowfin, unassociated / non FAD set, tuna purse seine |  | Dec 2011 |  | Yes | 790 670.0 | 2015 | Yes |  |  | ✓ |  |
|  | Louisiana blue crab |  | Mar 2012 |  |  | 18 724.5 | 2015 | No |  | ✓ |  |  |
|  | Norway North East Arctic cold water prawn |  | Mar 2012 |  |  | 10 234.0 | 2015 | No |  |  | ✓ |  |
|  | SSMO Shetland inshore brown crab and scallop |  | Mar 2012 |  |  | 749.5 | 2015 | No |  |  | ✓ |  |
|  | CSHMAC Celtic Sea herring trawl |  | Mar 2012 |  |  | 12 921.3 | 2016 | No | ✓ |  |  |  |
|  | Heard Island and McDonald Islands (HIMI) Toothfish |  | Mar 2012 |  |  | 3 144.0 | 2016 | No |  |  | ✓ |  |
|  | Compagnie des Pêches Saint Malo and Euronor cod and haddock |  | Apr 2012 |  |  | 8 367.1 | 2015 | No |  |  | ✓ |  |
|  | North West Atlantic Canada longline swordfish |  | Apr 2012 |  | Yes | 1 397.6 | 2016 | No |  |  | ✓ |  |
|  | Limfjord blue shell mussel (rope grown) |  | Apr 2012 |  |  | 1 245.0 | 2014 | No |  |  | ✓ |  |
|  | ISF Iceland Cod |  | Apr 2012 |  |  | 234 722.0 | 2015 | Yes |  |  | ✓ |  |
|  | ISF Iceland haddock |  | Apr 2012 |  |  | 37 153.0 | 2015 | Yes |  |  | ✓ |  |
|  | New Zealand southern blue whiting trawl |  | Apr 2012 |  |  | 32 955.0 | 2014 | Yes |  |  | ✓ |  |
|  | DFPO Limfjord oyster dredge |  | May 2012 |  |  | 81.0 | 2014 | No |  |  | ✓ |  |
|  | Argentine hoki (*Macruronus magellanicus*) bottom and mid-water trawl |  | May 2012 |  |  | 25 287.2 | 2016 | Yes |  |  | ✓ |  |
|  | Macquarie Island (MI) toothfish |  | May 2012 |  |  | 413.0 | 2015 | No |  |  | ✓ |  |
|  | DFPO Denmark North Sea sole |  | Jun 2012 |  |  | 277.0 | 2015 | No |  |  | ✓ |  |
|  | Shetland & Scottish Mainland rope grown mussel enhanced fishery |  | Jun 2012 |  |  | 6 392.0 | 2015 | No |  |  | ✓ |  |
|  | Dee Estuary cockle |  | Jul 2012 |  |  | 1 324.0 | 2016 | No |  |  | ✓ |  |
|  | Scotian Shelf snow crab trap |  | Jul 2012 |  |  | 11 913.0 | 2015 | No |  |  | ✓ |  |
|  | Clearwater Seafoods Banquereau and Grand Bank Arctic surf clam hydraulic dredge |  | Jul 2012 |  |  | 21 951.0 | 2015 | No | ✓ |  |  |  |
|  | DFPO Denmark North Sea & Skagerrak haddock |  | Aug 2012 |  |  | 2 337.7 | 2015 | No |  |  | ✓ |  |
|  | Faroe Islands silver smelt |  | Aug 2012 |  |  | 11 692.0 | 2016 | No |  |  | ✓ |  |
|  | US Atlantic spiny dogfish |  | Aug 2012 |  |  | 11 480.0 | 2016 | No |  |  | ✓ |  |
|  | Gulf of St Lawrence snow crab trap |  | Sep 2012 |  |  | 25 890.0 | 2015 | Yes |  |  | ✓ |  |
|  | OHV Dutch Waddenzee and Oosterschelde Hand Raked cockle |  | Oct 2012 |  |  | 5 927.0 | 2016 | No |  |  | ✓ |  |
|  | Australia Northern prawn |  | Nov 2012 |  |  | 10 063.0 | 2015 | No |  |  | ✓ |  |
|  | DFA Dutch North Sea ensis |  | Nov 2012 |  |  | 3 854.0 | 2016 | No |  |  | ✓ |  |
|  | Maldives pole & line tuna |  | Nov 2012 |  |  | 85 782.0 | 2016 | Yes |  |  | ✓ |  |
|  | CVO North Sea plaice and sole |  | Dec 2012 |  | Yes | 9 218.0 | 2016 | No |  |  | ✓ |  |
|  | West Greenland coldwater prawn |  | Feb 2013 |  |  | 82 000.0 | 2016 | Yes |  |  | ✓ |  |
|  | Dutch Oyster Association oyster |  | Feb 2013 |  |  | 3 264.0 | 2016 | No |  |  | ✓ |  |
|  | US North Atlantic swordfish |  | Mar 2013 |  |  | 1 258.2 | 2016 | No | ✓ |  |  |  |
|  | Newfoundland & Labrador snow crab |  | Apr 2013 |  |  | 37 153.0 | 2016 | Yes |  |  | ✓ |  |
|  | Japanese scallop hanging and seabed enhanced fisheries |  | May 2013 |  |  | 279 823.0 | 2016 | Yes |  |  | ✓ |  |
|  | Canada Atlantic halibut |  | May 2013 |  |  | 2 006.0 | 2015 | No |  |  | ✓ |  |
|  | FIUN Barents & Norwegian Seas cod and haddock |  | Jun 2013 |  |  | 272 800.0 | 2016 | Yes |  |  | ✓ |  |
|  | Iles-de-la-Madeleine lobster |  | Jul 2013 |  |  | 2 558.0 | 2016 | No |  |  | ✓ |  |
|  | FBSA Canada Full Bay sea scallop |  | Jul 2013 |  |  | 1 590.0 | 2015 | No |  |  | ✓ |  |
|  | Ireland bottom grown mussel |  | Jul 2013 |  |  | 10 500.0 | 2015 | No |  |  | ✓ |  |
|  | Northern Ireland bottom grown mussel |  | Jul 2013 |  |  | 1 639.7 | 2015 | No |  |  | ✓ |  |
|  | SARPC Toothfish |  | Aug 2013 |  |  | 6 973.0 | 2016 | No |  |  | ✓ |  |
|  | Russia Sea of Okhotsk pollock |  | Sep 2013 |  | Yes | 751 952.0 | 2016 | Yes |  |  | ✓ |  |
|  | Scottish Fisheries Sustainable Accreditation Group (SFSAG) saithe |  | Oct 2013 |  |  | 10 671.0 | 2014 | No |  |  | ✓ |  |
|  | Germany Lower Saxony mussel dredge and mussel culture |  | Oct 2013 |  | Yes | 2 127.0 | 2016 | No |  |  | ✓ |  |
|  | Estonia North East Arctic cold water prawn |  | Nov 2013 |  |  | 7 066.0 | 2015 | No |  |  | ✓ |  |
|  | AGARBA Spain Barents Sea cod |  | Nov 2013 |  |  | 6 085.4 | 2016 | No |  |  | ✓ |  |
|  | Faroe Islands North East Arctic cold water prawn |  | Dec 2013 |  |  | 4 219.0 | 2014 | No |  |  | ✓ |  |
|  | US Atlantic sea scallop |  | Dec 2013 |  | Yes | 11 329.0 | 2014 | No |  |  | ✓ |  |
|  | SSPO Swedish West Coast rope grown mussel |  | Feb 2014 |  |  | 1 362.0 | 2016 | No |  |  | ✓ |  |
|  | Chilean mussel fishery and suspended culture Toralla S.A and Cultivos Toralla S.A |  | Feb 2014 |  |  | 7 241.4 | 2017 | No |  | ✓ |  |  |
|  | Falkland Island toothfish |  | Mar 2014 |  | Yes | 1 123.0 | 2016 | No |  |  | ✓ |  |
|  | AAFA and WFOA North Pacific albacore tuna |  | Apr 2014 |  |  | 9 500.0 | 2016 | No | ✓ |  |  |  |
|  | AAFA and WFOA South Pacific albacore tuna |  | Apr 2014 |  |  | 145.0 | 2016 | No |  | ✓ |  |  |
|  | ISF Norwegian & Icelandic herring trawl and seine |  | May 2014 |  |  | 68 952.0 | 2015 | Yes |  |  | ✓ |  |
|  | US West Coast limited entry groundfish trawl |  | Jun 2014 |  |  | 63 630.0 | 2016 | Yes |  | ✓ |  |  |
|  | Waterhen Lake walleye and Northern pike gillnet |  | Jun 2014 |  |  | 27.9 | 2017 | No |  | ✓ |  |  |
|  | Northern Ireland Pelagic Sustainability Group (NIPSG) Irish Sea herring |  | Aug 2014 |  |  | 5 074.6 | 2015 | No |  |  | ✓ |  |
|  | ISF Iceland golden redfish, blue ling and tusk |  | Sep 2014 |  | Yes | 48 078.0 | 2017 | Yes |  |  | ✓ |  |
|  | ISF Iceland saithe, ling, Atlantic wolffish and plaice |  | Sep 2014 |  |  | 52 422.0 | 2017 | Yes |  |  | ✓ |  |
|  | New Zealand EEZ ling trawl and longline |  | Sep 2014 |  |  | 14 167.0 | 2014 | No |  |  | ✓ |  |
|  | New Zealand hake trawl |  | Sep 2014 |  |  | 2 415.0 | 2016 | No |  |  | ✓ |  |
|  | NAFO Division 4R Atlantic herring purse seine |  | Oct 2014 |  |  | 11 384.0 | 2015 | No |  | ✓ |  |  |
|  | DFPO Denmark North Sea, Skagerrak and Kattegat hake and plaice |  | Oct 2014 |  |  | 21 715.0 | 2016 | No |  | ✓ |  |  |
|  | Ashtamudi Estuary short-necked clam |  | Nov 2014 |  |  | 10 368.0 | 2016 | No |  |  | ✓ |  |
|  | Prince Edward Island lobster trap |  | Nov 2014 |  |  | 12 943.1 | 2016 | No |  | ✓ |  |  |
|  | Icelandic Gillnet lumpfish |  | Dec 2014 |  |  | 5 414.0 | 2016 | No |  |  | ✓ |  |
|  | Juan Fernández Rock lobster |  | Jan 2015 |  |  | 106.2 | 2017 | No |  | ✓ |  |  |
|  | Danish and Swedish nephrops |  | Jan 2015 |  |  | 1 674.0 | 2016 | No |  |  | ✓ |  |
|  | Gaspésie lobster trap |  | Mar 2015 |  |  | 1 926.3 | 2016 | No |  | ✓ |  |  |
|  | Cantabrian Sea purse seine anchovy |  | Mar 2015 |  |  | 8 139.6 | 2016 | No |  | ✓ |  |  |
|  | Faroese Pelagic Organisation North East Atlantic mackerel |  | Mar 2015 |  |  | 87 500.0 | 2014 | Yes |  |  | ✓ |  |
|  | Western Baltic spring spawning herring |  | Apr 2015 |  |  | 9 075.0 | 2016 | No |  | ✓ |  |  |
|  | FROM Nord North Sea and Eastern Channel pelagic trawl herring |  | Apr 2015 |  |  | 5 069.0 | 2015 | No |  |  | ✓ |  |
|  | Zhangzidao scallop |  | Apr 2015 |  |  | 19 217.7 | 2016 | No |  | ✓ |  |  |
|  | Greenland cod, haddock and saithe trawl |  | May 2015 |  | Yes | 10 540.0 | 2017 | No |  | ✓ |  |  |
|  | Bay of Fundy, Scotian Shelf and Southern Gulf of St. Lawrence lobster trap |  | May 2015 |  |  | 71 748.0 | 2016 | Yes |  | ✓ |  |  |
|  | NKFPO Bothnian Bay vendace trawl |  | Jun 2015 |  |  | 1 746.0 | 2015 | No |  |  | ✓ |  |
|  | SZLC, CSFC & FZLC Cook Islands EEZ South Pacific albacore & yellowfin longline |  | Jun 2015 |  | Yes | 4 667.0 | 2015 | No |  |  | ✓ |  |
|  | Cornish hake gill net |  | Jun 2015 |  |  | 1 093.5 | 2016 | No |  | ✓ |  |  |
|  | Greenland lumpfish |  | Aug 2015 |  | Yes | 7 396.8 | 2017 | No |  | ✓ |  |  |
|  | Lake Erie multi-species |  | Aug 2015 |  |  | 2 565.0 | 2015 | No |  |  | ✓ |  |
|  | Australia blue grenadier |  | Aug 2015 |  |  |  | 2016 | No |  | ✓ |  |  |
|  | Sweden Skagerrak, Kattegat and the Norwegian Deep cold-water prawn |  | Oct 2015 |  |  | 2 095.0 | 2016 | No |  | ✓ |  |  |
|  | Exmouth Gulf Prawns |  | Oct 2015 |  |  | 496.0 | 2017 | No |  | ✓ |  |  |
|  | Shark Bay prawn |  | Oct 2015 |  |  | 1 606.0 | 2017 | No |  | ✓ |  |  |
|  | Gulf of St Lawrence fall herring gillnet |  | Nov 2015 |  |  | 4 531.0 | 2016 | No |  | ✓ |  |  |
|  | DFPO Limfjord mussel and cockle |  | Dec 2015 |  | Yes | 22 900.0 | 2014 | No |  |  | ✓ |  |
|  | SPSG, DPPO, PFA, SPFPO & KFO Atlanto-Scandian purse seine and pelagic trawl herring |  | Jan 2016 |  |  | 22 180.4 | 2016 | No |  | ✓ |  |  |
|  | Arkhangelsk Trawl fleet Norwegian & Barents Seas cod, haddock & saithe |  | Jan 2016 |  |  | 36 602.7 | 2016 | Yes |  |  | ✓ |  |
|  | PFA, DPPO, KFO, SPSG & Compagnie des Pêches St Malo Northeast Atlantic blue whiting pelagic trawl |  | Feb 2016 |  |  | 151 265.9 | 2016 | Yes |  | ✓ |  |  |
|  | Western Asturias octopus traps fishery of artisanal Cofradias |  | Feb 2016 |  |  | 34.0 | 2015 | No |  |  | ✓ |  |
|  | Irikla Reservoir perch |  | Apr 2016 |  |  | 212.9 | 2016 | No |  | ✓ |  |  |
|  | Denmark Skagerrak and the Norwegian Deep cold-water prawn |  | May 2016 |  |  | 1 997.0 | 2016 | No |  | ✓ |  |  |
|  | FROM Nord North Sea and Eastern Channel trammel net sole |  | May 2016 |  |  | 972.0 | 2016 | No |  |  | ✓ |  |
|  | MINSA North East Atlantic mackerel |  | May 2016 |  |  | 584 616.7 | 2016 | Yes |  | ✓ |  |  |
|  | Bratsk Reservoir perch |  | May 2016 |  |  | 921.4 | 2016 | No |  |  | ✓ |  |
|  | Tri Marine Western and Central Pacific skipjack and yellowfin tuna |  | Jun 2016 |  |  | 23 455.0 | 2016 | No |  | ✓ |  |  |
|  | North Atlantic albacore artisanal |  | Jun 2016 |  |  | 4 300.0 | 2016 | No |  |  | ✓ |  |
|  | Norway Skagerrak and the Norwegian deep cold water prawn |  | Jun 2016 |  |  | 5 400.0 | 2014 | No |  |  | ✓ |  |
|  | Faroese Pelagic Organization North East Atlantic blue whiting |  | Jun 2016 |  |  | 211 647.0 | 2014 | Yes |  |  | ✓ |  |
|  | Peel Harvey estuarine fishery: recreational and commercial blue swimmer crab and commercial sea mull |  | Jun 2016 |  |  | 114.0 | 2015 | No |  |  | ✓ |  |
|  | Cancale Bay slipper limpet dredge |  | Jul 2016 |  |  | 400.0 | 2015 | No |  |  | ✓ |  |
|  | Solomon Islands skipjack and yellowfin tuna purse seine and pole and line |  | Jul 2016 |  |  | 26 745.0 | 2015 | Yes |  | ✓ |  |  |
|  | Australian West Coast deep sea crab |  | Jul 2016 |  |  | 153.3 | 2016 | No |  |  | ✓ |  |
|  | Chile squat lobsters and nylon shrimp modified trawl |  | Sep 2016 |  |  | 14 300.0 | 2014 | No | ✓ |  |  |  |
|  | VA-Delta Kamchatka salmon fisheries |  | Sep 2016 |  |  | 3 132.0 | 2015 | No |  |  | ✓ |  |
|  | DFPO, DPPO and SPFPO Skagerrak, Kattegat and Western Baltic herring fishery |  | Oct 2016 |  |  | 4 244.0 | 2014 | No |  |  | ✓ |  |
|  | Southern Gulf of California thread herring |  | Oct 2016 |  |  | 82 424.0 | 2016 | Yes |  | ✓ |  |  |
|  | Japanese pole and line skipjack and albacore tuna |  | Oct 2016 |  |  | 3 460.0 | 2016 | No |  |  | ✓ |  |
|  | Schleswig-Holstein blue shell mussel |  | Oct 2016 |  |  | 3 427.0 | 2014 | No |  |  | ✓ |  |
|  | South Australia Lakes and Coorong pipi |  | Nov 2016 |  |  | 299.0 | 2011 | No | ✓ |  |  |  |
|  | Canadian 4VWX purse seine herring |  | Nov 2016 |  |  | 48 528.0 | 2016 | Yes |  |  | ✓ |  |
|  | Northern Ireland Pelagic Sustainability Group (NIPSG) Irish Sea-Atlantic mackerel & North Sea herring |  | Dec 2016 |  |  | 21 509.0 | 2014 | No |  |  | ✓ |  |
|  | New Zealand orange roughy |  | Dec 2016 |  | Yes | 5 582.0 | 2017 | No |  | ✓ |  |  |
|  | Gulf of Maine lobster |  | Dec 2016 |  |  | 54 884.7 | 2015 | Yes | ✓ |  |  |  |
|  | US Atlantic surfclam and ocean quahog |  | Dec 2016 |  |  | 33 100.0 | 2014 | Yes |  |  | ✓ |  |
|  | Bay of Biscay purse seine sardine |  | Jan 2017 |  |  | 2 566.8 | 2015 | No |  |  | ✓ |  |
|  | Chile squat lobsters demersal trawl Camanchaca Fishery |  | Feb 2017 |  |  | 2 882.0 | 2015 | No |  |  | ✓ |  |
|  | PFA & SPSG North Sea herring |  | Apr 2017 |  |  | 198 973.0 | 2015 | Yes |  |  | ✓ |  |
|  | ISF Iceland capelin |  | Apr 2017 |  |  | 353 713.0 | 2015 | Yes |  |  | ✓ |  |
|  | Western Australia abalone |  | Apr 2017 |  |  | 242.0 | 2014 | No |  |  | ✓ |  |
|  | British Columbia salmon |  | Apr 2017 |  |  | 40 909.8 | 2014 | Yes |  |  | ✓ |  |
|  | DFPO Inner Danish Waters blue shell mussel |  | May 2017 |  |  | 24 330.0 | 2016 | No |  |  | ✓ |  |
|  | Canada 3LN redfish |  | May 2017 |  |  | 14 000.0 | 2014 | No |  |  | ✓ |  |
|  | LFPO pelagic trawl sprat (*Sprattus sprattus*) |  | May 2017 |  |  | 16 437.0 | 2016 | No |  |  | ✓ |  |
|  | West Greenland offshore Greenland halibut |  | May 2017 |  |  | 13 700.0 | 2015 | No |  |  | ✓ |  |
|  | SIC Lake Mälaren and Lake Vänern pikeperch |  | Jul 2017 |  |  | 261.0 | 2014 | No |  |  | ✓ |  |
|  | Scottish Fisheries Sustainable Accreditation Group (SFSAG) North Sea cod |  | Jul 2017 |  |  | 13 246.0 | 2015 | No |  |  | ✓ |  |
|  | Talley’s New Zealand Skipjack Tuna Purse Seine |  | Aug 2017 |  |  | 3 888.0 | 2015 | No | ✓ |  |  |  |
|  | Australia silver lipped pearl oyster |  | Sep 2017 |  |  | 112 917.0 | 2014 | Yes |  |  | ✓ |  |
|  | Northeastern Tropical Pacific purse seine yellowfin and skipjack tuna |  | Sep 2017 |  |  | 132 586.0 | 2014 | Yes |  |  | ✓ |  |
|  | Basse Normandy Granville Bay whelk |  | Sep 2017 |  |  | 6 000.0 | 2015 | No |  |  | ✓ |  |
|  | NFA Norwegian Ling & Tusk and NFA Norwegian Lumpfish |  | Oct 2017 |  |  | 30 313.0 | 2016 | Yes | ✓ |  |  |  |
|  | ISF Iceland mackerel |  | Oct 2017 |  |  | 170 516.0 | 2016 | Yes |  |  | ✓ |  |
|  | Lake Peipus perch and pike-perch |  | Oct 2017 |  |  | 716.3 | 2016 | No | ✓ |  |  |  |
|  | ISF Greenland halibut |  | Oct 2017 |  |  | 13 497.0 | 2016 | No | ✓ |  |  |  |
|  | American Samoa EEZ albacore and yellowfin longline |  | Nov 2017 |  |  | 1 806.0 | 2016 | No | ✓ |  |  |  |
|  | North Sea brown shrimp |  | Dec 2017 |  |  | 30 454.0 | 2015 | Yes |  |  | ✓ |  |
| Certified with component(s) in assessment | Norway North Sea demersal |  | Jun 2008 |  |  | 36 945.0 | 2015 | Yes |  |  | ✓ |  |
|  | Gulf of St Lawrence northern shrimp trawl Esquiman Channel |  | Mar 2009 |  |  | 28 010.0 | 2016 | Yes |  | ✓ |  |  |
|  | Norway spring spawning herring |  | Apr 2009 |  |  | 172 638.0 | 2015 | Yes |  |  | ✓ |  |
|  | Osprey Trawlers North Sea twin-rigged plaice |  | Sep 2010 |  |  | 1 931.0 | 2014 | No |  |  | ✓ |  |
|  | Scottish Fisheries Sustainable Accreditation Group (SFSAG) North Sea haddock |  | Oct 2010 |  |  | 72 772.0 | 2016 | Yes | ✓ |  |  |  |
|  | UK Fisheries Ltd/DFFU/Doggerbank Northeast Arctic cod, haddock and saithe |  | May 2012 |  |  | 25 388.0 | 2015 | Yes |  |  | ✓ |  |
|  | Fiji albacore tuna longline |  | Dec 2012 |  |  | 1 417.3 | 2014 | No |  |  | ✓ |  |
|  | Russian Federation Barents sea cod and haddock |  | May 2014 |  |  | 20 474.6 | 2015 | No |  |  | ✓ |  |
|  | Walker Seafood Australian albacore, yellowfin tuna, and swordfish longline |  | Aug 2015 |  |  | 929.7 | 2014 | No |  |  | ✓ |  |
|  | US Acadian redfish, haddock and pollock otter trawl |  | Jul 2016 |  |  | 9 447.9 | 2014 | No |  |  | ✓ |  |
|  | DFPO and DPPO North Sea, Skagerrak and Kattegat sandeel, sprat and Norway pout |  | Mar 2017 |  |  | 320 895.0 | 2014 | Yes |  |  | ✓ |  |
|  | Faroe Islands and Iceland North East Arctic cod, haddock and saithe |  | Aug 2017 |  |  | 36 485.8 | 2015 | Yes |  |  | ✓ |  |
| Combined | Pelagic Freezer Trawler Association North Sea herring | Combined as PFA & SPSG North Sea herring | May 2006 | Apr 2017 |  | 149 924.0 | 2014 | Yes |  |  | ✓ |  |
|  | American Albacore Fishing Association (AAFA) North Pacific albacore pole & line and troll/Jig | Combined as AAFA and WFOA North Pacific albacore tuna | Aug 2007 | Apr 2014 |  | 5 432.3 | 2010 | No |  | ✓ |  |  |
|  | SPSG Ltd North Sea herring | Combined as PFA & SPSG North Sea herring | Jul 2008 | Apr 2017 |  | 51 595.0 | 2014 | Yes |  |  | ✓ |  |
|  | Danish Pelagic Producers Organisation Atlanto Scandian herring | Combined as SPSG, DPPO, PFA, SPFPO & KFO Atlanto-Scandian purse seine and pelagic trawl herring | Jul 2009 | Jan 2016 |  | 13 806.0 | 2013 | No |  | ✓ |  |  |
|  | SPSG Ltd Atlanto Scandian herring |  | Mar 2010 | Jan 2016 |  | 8 342.0 | 2013 | No |  | ✓ |  |  |
|  | Euronor saithe | Combined as Scapeche, Euronor and Compagnie de Peche de St Malo saith | Mar 2010 | Sep 2016 |  | 11 454.0 | 2013 | No |  | ✓ |  |  |
|  | American Western Fish Boats Owners Association (WFOA) albacore tuna North Pacific | Combined as AAFA and WFOA North Pacific albacore tuna | Mar 2010 | Apr 2014 |  | 5 432.3 | 2010 | No |  | ✓ |  |  |
|  | British Columbia sockeye salmon | Combined as British Columbia Salmon | Jul 2010 | Apr 2017 | Yes | 1 857.0 | 2012 | No |  |  | ✓ |  |
|  | Pelagic Freezer-Trawler Association Atlanto-Scandian herring | Combined as SPSG, DPPO, PFA, SPFPO & KFO Atlanto-Scandian purse seine and pelagic trawl herring | Jul 2010 | Jan 2016 |  | 9 671.0 | 2013 | No |  | ✓ |  |  |
|  | Scapêche and Compagnie des Pêches Saint Malo saithe | Combined as Scapeche, Euronor and Compagnie de Peche de St Malo saith | Jan 2011 | Sep 2016 |  | 1 846.2 | 2014 | No |  |  | ✓ |  |
|  | British Columbia pink salmon | Combined as British Columbia Salmon | Jul 2011 | Apr 2017 |  | 1 203.0 | 2012 | No |  |  | ✓ |  |
|  | Isefjord and East Jutland Danish blue shell mussel | Combined as DFPO Inner Danish Waters blue shell mussel | Jan 2012 | May 2017 | Yes | 12 055.0 | 2014 | No |  |  | ✓ |  |
|  | Vilsund Blue East Jutland blue shell mussel dredge |  | Mar 2012 | May 2017 |  | 3 800.0 | 2014 | No |  |  | ✓ |  |
|  | Seafood Romo East Jutland and Isefjord blue shell mussel dredge |  | Mar 2012 | May 2017 |  | 448.0 | 2015 | No |  | ✓ |  |  |
|  |  |  |  |  |  |  |  |  |  |  |  |  |
|  | Faroe Islands North East Arctic cod and saithe | Combined as Faroe Islands and Iceland North East Arctic cod, haddock and saithe | Aug 2012 | Aug 2017 |  | 36 485.8 | 2015 | Yes |  |  | ✓ |  |
|  | Faroe Islands North East Arctic haddock |  | Aug 2012 | Aug 2017 |  | 2 652.9 | 2015 | No |  |  | ✓ |  |
|  | British Columbia chum salmon | Combined as British Columbia Salmon | Jan 2013 | Apr 2017 |  | 3 000.0 | 2012 | No |  |  | ✓ |  |
|  | Faroe Islands saithe | Combined as Faroe Islands and Iceland North East Arctic cod, haddock and saithe | Jun 2013 | Aug 2017 |  | 12 332.3 | 2015 | No |  |  | ✓ |  |
| Split | Lakes and Coorong, South Australia | Split in two fisheries, thus considered 'combined' at the date the first of the two was certified | Jun 2008 | Nov 2016 |  | 937.0 | 2011 | No |  | ✓ |  |  |
| Suspended | Kyoto Danish Seine Fishery Federation flathead flounder |  | Sep 2008 | Nov 2017 |  | 77.0 | 2016 | No |  | ✓ |  |  |
|  | SPSG Ltd western component of north east Atlantic mackerel |  | Jan 2009 | Mar 2012 |  | 149 497.0 | 2012 | Yes |  | ✓ |  |  |
|  | North East Atlantic mackerel pelagic trawl, purse-seine and handline |  | Apr 2009 | Mar 2012 |  | 176 017.0 | 2012 | Yes |  | ✓ |  |  |
|  | Irish Pelagic Sustainability Group (IPSG) western mackerel pelagic trawl |  | Aug 2009 | Mar 2012 |  | 53 721.5 | 2012 | Yes |  | ✓ |  |  |
|  | Irish Pelagic Sustainability Association (IPSA) western mackerel |  | Jul 2010 | Mar 2012 |  | 5 800.0 | 2013 | No |  | ✓ |  |  |
|  | SPFPO North East Atlantic mackerel |  | Sep 2011 | Mar 2012 | Yes | 3 250.0 | 2011 | No |  | ✓ |  |  |
|  | SPSG West of Scotland herring pelagic trawl |  | Apr 2012 | Feb 2016 |  | 10 654.0 | 2016 | No |  |  | ✓ |  |
|  | Northeast Sakhalin Island pink salmon trap net |  | Jun 2012 | Jun 2015 |  | 5 170.0 | 2013 | No |  | ✓ |  |  |
|  | Razor clam fishery from Ria de Pontevedra |  | Jan 2013 | Jan 2014 |  | 60.0 | 2015 | No |  |  | ✓ |  |
|  | Maine lobster trap |  | Mar 2013 | Aug 2017 |  | 56 099.0 | 2016 | Yes |  |  | ✓ |  |
|  | Clams and cockle fishery from Ria de Arousa |  | May 2013 | May 2014 |  | 20.0 | 2012 | No |  |  | ✓ |  |
|  | Faroe Islands queen scallop |  | Sep 2013 | Jan 2017 |  | 5 061.0 | 2015 | No |  |  | ✓ |  |
|  | Grupo Regal Spain hake longline |  | Apr 2014 | Aug 2016 |  | 1 800.0 | 2015 | No |  |  | ✓ |  |
|  | Companhia de Pescarias do Algarve rope grown Mediterranean mussel |  | Nov 2014 | Aug 2016 |  | 210.0 | 2016 | No |  |  | ✓ |  |
|  | Poland Eastern Baltic cod |  | Jan 2015 | Dec 2015 |  | 8 934.0 | 2013 | No |  |  | ✓ |  |
|  | LFA Latvia trawl eastern Baltic cod |  | Jul 2015 | Dec 2015 |  | 5 500.0 | 2013 | No |  |  | ✓ |  |
|  | Rimfrost Antarctic krill |  | Aug 2015 | Jun 2017 |  | 11 029.0 | 2015 | No |  | ✓ |  |  |
|  | Canada/Newfoundland 3Ps cod |  | Mar 2016 | May 2017 |  | 4 031.0 | 2014 | No |  |  | ✓ |  |
| Withdrawn | Thames Blackwater herring drift-net |  | Mar 2000 | Dec 2009 |  | 2.0 | 2007 | No |  | ✓ |  |  |
|  | South West handline mackerel |  | Aug 2001 | Feb 2012 |  | 820.0 | 2010 | No |  | ✓ |  |  |
|  | Loch Torridon nephrops creel |  | Jan 2003 | Jan 2011 |  | 100.0 | 2006 | No | ✓ |  |  |  |
|  | Hastings fleet pelagic herring |  | Sep 2005 | Dec 2016 |  | 10.0 | 2012 | No |  |  | ✓ |  |
|  | Bering Sea and Aleutian Island Alaska (Pacific) cod freezer longline |  | Feb 2006 | Jan 2010 |  | 106 832.0 | 2007 | Yes |  | ✓ |  |  |
|  | North Eastern Inshore Fisheries and Conservation Authority sea bass |  | Sep 2007 | Dec 2011 |  | 10.6 | 2010 | No |  | ✓ |  |  |
|  | Domstein Longliner Partners North East Arctic cod |  | Feb 2009 | Nov 2011 |  | 5 000.0 | 2007 | No | ✓ |  |  |  |
|  | Domstein Longliner Partners North East Arctic haddock |  | Feb 2009 | Nov 2011 |  | 2 500.0 | 2007 | No | ✓ |  |  |  |
|  | Stornoway nephrops trawl |  | Mar 2009 | Mar 2012 |  | 567.3 | 2011 | No |  | ✓ |  |  |
|  | Pelagic Freezer Trawler Association North East Atlantic mackerel pelagic trawl |  | Jul 2009 | Mar 2012 |  | 67 953.0 | 2012 | Yes |  | ✓ |  |  |
|  | Danish Pelagic Producers Organisation North East Atlantic mackerel |  | Jul 2009 | Mar 2012 |  | 24 024.0 | 2009 | No | ✓ |  |  |  |
|  | Atlantic deep sea red crab |  | Sep 2009 | Sep 2014 |  | 1 180.0 | 2012 | No |  | ✓ |  |  |
|  | Tosakatsuo Suisan skipjack tuna |  | Nov 2009 | Nov 2012 |  | 15 057.0 | 2010 | No |  | ✓ |  |  |
|  | CVO sole gillnet |  | Nov 2009 | Nov 2013 |  | 76.0 | 2011 | No |  | ✓ |  |  |
|  | Vilsund Blue a/s Limfjord mussel & cockle dredge |  | Jan 2010 | Feb 2017 |  | 21 723.0 | 2015 | No |  | ✓ |  |  |
|  | Portugal sardine purse seine |  | Jan 2010 | Jan 2012 |  | 63 727.0 | 2010 | Yes |  | ✓ |  |  |
|  | Canada sablefish |  | Jul 2010 | Aug 2013 |  | 1 836.5 | 2012 | No |  | ✓ |  |  |
|  | Oregon Dungeness crab |  | Dec 2010 | Jan 2015 |  | 6 545.3 | 2014 | No |  | ✓ |  |  |
|  | DFPO Denmark Eastern Baltic cod |  | Apr 2011 | Dec 2015 |  | 5 286.0 | 2014 | No |  | ✓ |  |  |
|  | Isle of Man Queen scallop trawl |  | May 2011 | May 2014 |  | 3 979.0 | 2012 | No |  |  | ✓ |  |
|  | SPPO North Sea herring |  | May 2011 | Jul 2013 |  | 8 086.0 | 2011 | No |  | ✓ |  |  |
|  | Scotian Shelf shrimp |  | Jun 2011 | Feb 2014 |  | 4 100.0 | 2012 | No |  | ✓ |  |  |
|  | Swedish Fishermen’s Producer Organisation (SFPO) Eastern Baltic cod |  | Jun 2011 | Dec 2015 |  | 4 434.0 | 2014 | No |  | ✓ |  |  |
|  | Atlantic cod and haddock longline, handline and Danish seine |  | Jun 2011 | Jun 2013 |  | 12 887.4 | 2010 | No | ✓ |  |  |  |
|  | Small Pelagics Fishery in Sonora, Gulf of California |  | Jul 2011 | Jul 2016 | Yes | 124 490.0 | 2016 | Yes |  |  | ✓ |  |
|  | Netherlands suspended culture mussel |  | Jul 2011 | Oct 2016 |  | 20 300.0 | 2015 | No |  |  | ✓ |  |
|  | Germany Eastern Baltic cod |  | Aug 2011 | Dec 2015 |  | 504.0 | 2014 | No |  | ✓ |  |  |
|  | British Columbia spiny dogfish |  | Sep 2011 | Sep 2016 |  | 380.0 | 2012 | No |  | ✓ |  |  |
|  | Küstenfischer Nord eG Heiligenhafen Germany Eastern Baltic cod |  | Oct 2011 | Apr 2014 |  | 758.5 | 2011 | No |  | ✓ |  |  |
|  | Fogo Island Co-operative Society Limited cold water shrimp |  | Oct 2011 | Jul 2012 |  | 3 929.0 | 2010 | No | ✓ |  |  |  |
|  | Southeast US North Atlantic swordfish |  | Dec 2011 | Mar 2013 | Yes | 1 695.7 | 2008 | No | ✓ |  |  |  |
|  | Dutch rod and line fishery for sea bass |  | Dec 2011 | Feb 2015 |  | 26.7 | 2014 | No |  | ✓ |  |  |
|  | Pescafria-Pesquera Rodriguez Barents Sea cod |  | Feb 2012 | Mar 2013 |  | 3 760.0 | 2010 | No | ✓ |  |  |  |
|  | Royal Frysk Jutland mussels |  | Mar 2012 | Apr 2015 |  | 3 912.0 | 2013 | No |  | ✓ |  |  |
|  | Mexico Baja California pole and line yellowfin and skipjack tuna |  | May 2012 | May 2015 |  | 389.4 | 2013 | No |  | ✓ |  |  |
|  | Exmouth mussels |  | Jul 2012 | Aug 2016 |  | 217.0 | 2015 | No |  |  | ✓ |  |
|  | Sian Ka'an and Banco Chinchorro Biosphere Reserves spiny lobster |  | Jul 2012 | Jun 2016 |  | 280.0 | 2012 | No |  |  | ✓ |  |
|  | Ozernaya River sockeye salmon |  | Sep 2012 | Oct 2017 |  | 17 000.0 | 2014 | No |  |  | ✓ |  |
|  | SSLLC US North Atlantic swordfish longline |  | Jun 2015 | Sep 2016 | Yes | 1 212.0 | 2011 | No |  |  | ✓ |  |
