# Supplementary material for: Small is beautiful, but large is certified: A comparison between fisheries the Marine Stewardship Council (MSC) features in its promotional materials and MSC-certified fisheries
Source: PLoS One. 2020 May 4;15(5):e0231073. doi: 10.1371/journal.pone.0231073 (PMC7197776; doi:10.1371/journal.pone.0231073)
Supplement: S2 Table — (DOCX) [file pone.0231073.s003.docx]

**S2 Table 2**. Summary of the pictures used in the analysis (since 2009).

| **Picture** | **Name of the fishery** | **Status** | **Country** | **Gear** | **Gear category** | **Scale** |
| --- | --- | --- | --- | --- | --- | --- |
| MSC_Facebook_1 | Unidentified fishery | - | Undetermined | Undetermined | Undetermined | Small scale |
| MSC_Facebook_2 | Unidentified fishery | - | Undetermined | Entangling nets | Passive gears | Small scale |
| MSC_Facebook_3 | Unidentified Gambian fishery | Never certified | Gambia | Undetermined (passive) | Passive gears | Small scale |
| MSC_Facebook_4 | Unidentified fishery | - | Undetermined | Undetermined | Undetermined | Small scale |
| MSC_Facebook_5 | Maldives pole & line tuna | Certified | Maldives | Other hooks & lines | Passive gears | Small scale |
| MSC_Facebook_6 | Unidentified Gambian fishery | Never certified | Gambia | Undetermined (passive) | Passive gears | Small scale |
| MSC_Facebook_7 | Maldives pole & line tuna | Certified | Maldives | Other hooks & lines | Passive gears | Large scale |
| MSC_Facebook_8 | Ashtamudi Estuary short-necked clam | Certified | India | Hand-operated gears | Passive gears | Small scale |
| MSC_Facebook_9 | South Africa hake trawl | Certified | South Africa | Bottom trawls & dredges | Active gears | Large scale |
| MSC_Facebook_10 | Unidentified fishery | - | Undetermined | Undetermined | Undetermined | Small scale |
| MSC_Facebook_11 | Unidentified Gambian fishery | Never certified | Gambia | Undetermined (passive) | Passive gears | Small scale |
| MSC_Facebook_12 | Ekofish Group-North Sea twin rigged otter trawl plaice | Certified | Netherlands | Bottom trawls & dredges | Active gears | Large scale |
| MSC_Facebook_13 | Maldives pole & line tuna | Certified | Maldives | Other hooks & lines | Passive gears | Large scale |
| MSC_Facebook_14 | Unidentified fishery | - | Undetermined | Undetermined | Active gears | Large scale |
| MSC_Facebook_15 | Unidentified fishery | - | Undetermined | Undetermined | Undetermined | Small scale |
| MSC_Facebook_16 | Alaska pollock - Bering Sea and Aleutian Islands | Certified | USA | Pelagic trawls | Active gears | Large scale |
| MSC_Facebook_17 | Vietnam Ben Tre clam hand gathered | Certified | Vietnam | Hand-operated gears | Passive gears | Small scale |
| MSC_Facebook_18 | Rimfrost Antarctic krill | Certified | Norway | Pelagic trawls | Active gears | Large scale |
| MSC_Facebook_19 | South Africa hake trawl | Certified | South Africa | Bottom trawls & dredges | Active gears | Large scale |
| MSC_Facebook_20 | CVO North Sea plaice and sole | Certified | Netherlands | Bottom trawls & dredges | Active gears | Large scale |
| MSC_Facebook_21 | Maldives pole & line tuna | Certified | Maldives | Other hooks & lines | Passive gears | Large scale |
| MSC_Facebook_22 | Maldives pole & line tuna | Certified | Maldives | Other hooks & lines | Passive gears | Large scale |
| MSC_Facebook_23 | Maldives pole & line tuna | Certified | Maldives | Other hooks & lines | Passive gears | Large scale |
| MSC_Facebook_24 | Unidentified fishery | - | Undetermined | Undetermined | Undetermined | Small scale |
| MSC_Facebook_25 | Alaska pollock - Bering Sea and Aleutian Islands | Certified | USA | Pelagic trawls | Active gears | Large scale |
| MSC_Facebook_26 | Russia Barents Sea red king crab | Certified | Russian Federation | Pots & traps | Passive gears | Large scale |
| MSC_Facebook_27 | Unidentified seaweed farming | - | Undetermined | Hand-operated gears | Passive gears | Small scale |
| MSC_Facebook_28 | Western Australia abalone fishery | Certified | Australia | Hand-operated gears | Passive gears | Small scale |
| MSC_Facebook_29 | Ashtamudi Estuary short-necked clam | Certified | India | Hand-operated gears | Passive gears | Small scale |
| MSC_Facebook_30 | Ashtamudi Estuary short-necked clam | Certified | India | Hand-operated gears | Passive gears | Small scale |
| MSC_Facebook_31 | Ashtamudi Estuary short-necked clam | Certified | India | Hand-operated gears | Passive gears | Small scale |
| MSC_Facebook_32 | Ashtamudi Estuary short-necked clam | Certified | India | Hand-operated gears | Passive gears | Small scale |
| MSC_Facebook_33 | Unidentified Dutch fishery | Certified | Netherlands | Undetermined (active) | Active gears | Large scale |
| MSC_Facebook_34 | Madagascar octopus fisheries | Never certified | Madagascar | Hand-operated gears | Passive gears | Small scale |
| MSC_Facebook_35 | Unidentified fishery | - | Undetermined | Undetermined (passive) | Passive gears | Small scale |
| MSC_Facebook_36 | Maldives pole & line tuna | Certified | Maldives | Other hooks & lines | Passive gears | Small scale |
| MSC_Facebook_37 | Peel Harvey estuarine fishery: recreational and commercial blue swimmer crab and commercial sea mull | Certified | Australia | Entangling nets | Passive gears | Small scale |
| MSC_Facebook_38 | Peel Harvey estuarine fishery: recreational and commercial blue swimmer crab and commercial sea mull | Certified | Australia | Entangling nets | Passive gears | Small scale |
| MSC_Facebook_39 | PNA Western and Central Pacific skipjack and yellowfin, unassociated / non FAD set, tuna purse seine | Certified | Undetermined | Purse seines | Active gears | Large scale |
| MSC_Facebook_40 | Madagascar octopus fisheries | Never certified | Madagascar | Hand-operated gears | Passive gears | Small scale |
| MSC_Facebook_41 | Maldives pole & line tuna | Certified | Maldives | Other hooks & lines | Passive gears | Small scale |
| MSC_Facebook_42 | Unidentified fishery | - | Undetermined | Entangling nets | Passive gears | Small scale |
| MSC_Facebook_43 | Unidentified fishery | - | Undetermined | Other hooks & lines | Passive gears | Small scale |
| MSC_Facebook_44 | Unidentified fishery | - | Undetermined | Other hooks & lines | Passive gears | Small scale |
| MSC_Facebook_45 | Peel Harvey estuarine fishery: recreational and commercial blue swimmer crab and commercial sea mull | Certified | Australia | Entangling nets | Passive gears | Small scale |
| MSC_Facebook_46 | Unidentified fishery | - | Undetermined | Undetermined | Undetermined | Large scale |
| MSC_Facebook_47 | Unidentified Chinese fishery | - | China | Undetermined | Undetermined | Small scale |
| MSC_Facebook_48 | US West Coast limited entry groundfish trawl | Certified | USA | Bottom trawls & dredges | Active gears | Large scale |
| MSC_Facebook_49 | US West Coast limited entry groundfish trawl | Certified | USA | Bottom trawls & dredges | Active gears | Large scale |
| MSC_Facebook_50 | Euglena Co., Ltd seaweed farming | - | Japan | Hand-operated gears | Passive gears | Small scale |
| MSC_Facebook_51 | Unidentified fishery | - | Undetermined | Other hooks & lines | Passive gears | Small scale |
| MSC_Facebook_52 | Unidentified fishery | - | Undetermined | Undetermined (passive) | Passive gears | Small scale |
| MSC_Facebook_53 | Unidentified fishery | - | Undetermined | Entangling nets | Passive gears | Small scale |
| MSC_Facebook_54 | Unidentified fishery | - | Undetermined | Entangling nets | Passive gears | Small scale |
| MSC_Facebook_55 | Euglena Co., Ltd seaweed farming | - | Japan | Hand-operated gears | Passive gears | Small scale |
| MSC_Facebook_56 | Unidentified Dutch fishery | Certified | Netherlands | Undetermined (active) | Active gears | Large scale |
| MSC_Facebook_57 | Lake Hjälmaren pikeperch fish-trap and gillnet | Certified | Sweden | Pots & traps | Passive gears | Small scale |
| MSC_Facebook_58 | Suriname Atlantic seabob shrimp | Certified | Suriname | Bottom trawls & dredges | Active gears | Large scale |
| MSC_Facebook_59 | Ashtamudi Estuary short-necked clam | Certified | India | Hand-operated gears | Passive gears | Small scale |
| MSC_Facebook_60 | Unidentified fishery | - | Undetermined | Pots & traps | Passive gears | Small scale |
| MSC_Facebook_61 | Madagascar octopus fisheries | Never certified | Madagascar | Hand-operated gears | Passive gears | Small scale |
| MSC_Facebook_62 | Madagascar octopus fisheries | Never certified | Madagascar | Hand-operated gears | Passive gears | Small scale  Small scale |
| MSC_Facebook_63 | Alaska salmon | Certified | USA | Purse seines | Active gears |  |
| MSC_Facebook_64 | Falkland Island toothfish | Certified | UK | Longlines | Passive gears | Large scale |
| MSC_Facebook_65 | Falkland Island toothfish | Certified | UK | Longlines | Passive gears | Large scale |
| MSC_Facebook_66 | Falkland Island toothfish | Certified | UK | Longlines | Passive gears | Large scale |
| MSC_Facebook_67 | Falkland Island toothfish | Certified | UK | Longlines | Passive gears | Large scale |
| MSC_Facebook_68 | Falkland Island toothfish | Certified | UK | Longlines | Passive gears | Large scale |
| MSC_Facebook_69 | Unidentified fishery | - | Undetermined | Entangling nets | Passive gears | Small scale |
| MSC_Facebook_70 | Lake Hjälmaren pikeperch fish-trap and gillnet | Certified | Sweden | Pots & traps | Passive gears | Small scale |
| MSC_Facebook_71 | Ashtamudi Estuary short-necked clam | Certified | India | Hand-operated gears | Passive gears | Small scale |
| MSC_Facebook_72 | Unidentified fishery | - | Undetermined | Undetermined (active) | Active gears | Large scale |
| MSC_Facebook_73 | US West Coast limited entry groundfish trawl | Certified | USA | Bottom trawls & dredges | Active gears | Large scale |
| MSC_Facebook_74 | South Africa hake trawl | Certified | South Africa | Bottom trawls & dredges | Active gears | Large scale |
| MSC_Facebook_75 | Unidentified fishery | - | Undetermined | Undetermined (passive) | Passive gears | Small scale |
| MSC_Facebook_76 | Unidentified fishery | - | Undetermined | Undetermined (passive) | Passive gears | Small scale |
| MSC_Facebook_77 | Rimfrost Antarctic krill | Certified | Norway | Pelagic trawls | Active gears | Large scale |
| MSC_Facebook_78 | Rimfrost Antarctic krill | Certified | Norway | Pelagic trawls | Active gears | Large scale |
| MSC_Facebook_79 | Rimfrost Antarctic krill | Certified | Norway | Pelagic trawls | Active gears | Large scale |
| MSC_Facebook_80 | Australian Western rock lobster | Certified | Australia | Pots & traps | Passive gears | Large scale |
| MSC_Facebook_81 | South Georgia Patagonian toothfish longline | Certified | Argentina | Longlines | Passive gears | Large scale |
| MSC_Facebook_82 | Unidentified fishery | - | Undetermined | Undetermined (passive) | Passive gears | Small scale |
| MSC_Facebook_83 | Ashtamudi Estuary short-necked clam | Certified | India | Hand-operated gears | Passive gears | Small scale |
| MSC_Facebook_84 | South Africa hake trawl | Certified | South Africa | Bottom trawls & dredges | Active gears | Large scale |
| MSC_Facebook_85 | Maldives pole & line tuna | Certified | Maldives | Other hooks & lines | Passive gears | Large scale |
| MSC_Facebook_86 | ISF Norwegian & Icelandic herring trawl and seine | Certified | Iceland | Bottom trawls & dredges | Active gears | Large scale |
| MSC_Facebook_87 | Unidentified fishery | - | Undetermined | Undetermined | Undetermined | Large scale |
| MSC_Facebook_88 | Unidentified fishery | - | Undetermined | Undetermined (passive) | Passive gears | Small scale |
| MSC_Facebook_89 | Unidentified fishery | - | Undetermined | Undetermined (active) | Active gears | Large scale |
| MSC_Facebook_90 | Maldives pole & line tuna | Certified | Maldives | Other hooks & lines | Passive gears | Large scale |
| MSC_Facebook_91 | Maldives pole & line tuna | Certified | Maldives | Other hooks & lines | Passive gears | Large scale |
| MSC_Facebook_92 | US North Pacific halibut | Certified | USA | Longlines | Passive gears | Large scale |
| MSC_Facebook_93 | Chile squat lobsters and nylon shrimp modified Trawl | Certified | Chile | Bottom trawls & dredges | Active gears | Large scale |
| MSC_Facebook_94 | Juan Fernández Rock lobster | Certified | Chile | Pots & traps | Passive gears | Small scale |
| MSC_Facebook_95 | Oregon Dungeness crab | Withdrawn | USA | Pots & traps | Passive gears | Small scale |
| MSC_Facebook_96 | Unidentified fishery | - | Undetermined | ENtangling nets | Passive gears | Small scale |
| MSC_Facebook_97 | Maldives pole & line tuna | Certified | Maldives | Other hooks & lines | Passive gears | Large scale |
| MSC_Facebook_98 | Germany Lower Saxony mussel dredge and mussel culture | Certified | Germany | Others | Passive gears | Undetermined |
| MSC_Facebook_99 | Lake Hjälmaren pikeperch fish-trap and gillnet | Certified | Sweden | Pots & traps | Passive gears | Small scale |
| MSC_Facebook_100 | Unidentified fishery | - | Undetermined | Undetermined (passive) | Passive gears | Small scale |
| MSC_Facebook_101 | Unidentified fishery | - | Undetermined | Bottom trawls & dredges | Active gears | Large scale |
| MSC_Facebook_102 | Compagnie des Pêches Saint Malo and Euronor cod and haddock | Certified | France | Bottom trawls & dredges | Active gears | Large scale |
| MSC_Facebook_103 | Unidentified Gambian fishery | Never certified | Gambia | Undetermined (passive) | Passive gears | Small scale |
| MSC_Reports_1 | South West handline mackerel | Withdrawn | UK | Other hooks & lines | Passive gears | Small scale |
| MSC_Reports_2 | South Africa hake trawl | Certified | South Africa | Bottom trawls & dredges | Active gears | Large scale |
| MSC_Reports_3 | AAFA and WFOA Pacific albacore tuna fisheries | Certified | USA | Other hooks & lines | Passive gears | Large scale |
| MSC_Reports_4 | US North Pacific sablefish | Certified | USA | Longlines | Passive gears | Large scale |
| MSC_Reports_5 | Domstein Longliner Partners North East Arctic fisheries | Withdrawn | Norway | Longlines | Passive gears | Large scale |
| MSC_Reports_6 | Alaska salmon | Certified | USA | Undetermined | Undetermined | Small scale |
| MSC_Reports_7 | Mexico Baja California red rock lobster | Certified | Mexico | Pots & traps | Passive gears | Small scale |
| MSC_Reports_8 | Pelagic Freezer Trawler Association North Sea herring | Certified | Europe | Pelagic trawls | Active gears | Large scale |
| MSC_Reports_9 | Australian Western rock lobster | Certified | Australia | Pots & traps | Passive gears | Large scale |
| MSC_Reports_10 | Australian Western rock lobster | Certified | Australia | Pots & traps | Passive gears | Large scale |
| MSC_Reports_11 | Thames Blackwater herring drift-net | Withdrawn | UK | Entangling nets | Passive gears | Small scale |
| MSC_Reports_12 | Thames Blackwater herring drift-net | Withdrawn | UK | Entangling nets | Passive gears | Small scale |
| MSC_Reports_13 | Alaska salmon | Certified | USA | Purse seines | Active gears | Large scale |
| MSC_Reports_14 | New Zealand hoki | Certified | New Zealand | Bottom trawls & dredges | Active gears | Large scale |
| MSC_Reports_15 | New Zealand hoki | Certified | New Zealand | Bottom trawls & dredges | Active gears | Large scale |
| MSC_Reports_16 | Burry Inlet cockles | Certified | UK | Hand-operated gears | Passive gears | Small scale |
| MSC_Reports_17 | Burry Inlet cockles | Certified | UK | Hand-operated gears | Passive gears | Small scale |
| MSC_Reports_18 | South West handline mackerel | Withdrawn | UK | Other hooks & lines | Passive gears | Small scale |
| MSC_Reports_19 | South West handline mackerel | Withdrawn | UK | Other hooks & lines | Passive gears | Small scale |
| MSC_Reports_20 | Loch Torridon nephrops creel | Withdrawn | UK | Pots & traps | Passive gears | Small scale |
| MSC_Reports_21 | Loch Torridon nephrops creel | Withdrawn | UK | Pots & traps | Passive gears | Small scale |
| MSC_Reports_22 | South Georgia Patagonian toothfish longline | Certified | Argentina | Longlines | Passive gears | Large scale |
| MSC_Reports_23 | South Georgia Patagonian toothfish longline | Certified | Argentina | Longlines | Passive gears | Large scale |
| MSC_Reports_24 | South Africa hake trawl | Certified | South Africa | Bottom trawls & dredges | Active gears | Large scale |
| MSC_Reports_25 | South Africa hake trawl | Certified | South Africa | Bottom trawls & dredges | Active gears | Large scale |
| MSC_Reports_26 | Mexico Baja California red rock lobster | Certified | Mexico | Pots & traps | Passive gears | Small scale |
| MSC_Reports_27 | Mexico Baja California red rock lobster | Certified | Mexico | Pots & traps | Passive gears | Small scale |
| MSC_Reports_28 | Alaska flatfish - Bering Sea and Aleutian Islands | Certified | USA | Bottom trawls & dredges | Active gears | Large scale |
| MSC_Reports_29 | Alaska flatfish - Bering Sea and Aleutian Islands | Certified | USA | Bottom trawls & dredges | Active gears | Large scale |
| MSC_Reports_30 | Hastings fleet Dover sole and Plaice | Certified | UK | Entangling nets | Passive gears | Small scale |
| MSC_Reports_31 | Hastings fleet Dover sole and Plaice | Certified | UK | Entangling nets | Passive gears | Small scale |
| MSC_Reports_32 | Bering Sea and Aleutian Island Alaska (Pacific) cod - freezer longline | Withdrawn | USA | Longlines | Passive gears | Large scale |
| MSC_Reports_33 | Bering Sea and Aleutian Island Alaska (Pacific) cod - freezer longline | Withdrawn | USA | Longlines | Passive gears | Large scale |
| MSC_Reports_34 | Australia mackerel icefish | Certified | Australia | Bottom trawls & dredges | Active gears | Large scale |
| MSC_Reports_35 | US North Pacific halibut | Certified | USA | Longlines | Passive gears | Undetermined |
| MSC_Reports_36 | US North Pacific halibut | Certified | USA | Longlines | Passive gears | Undetermined |
| MSC_Reports_37 | US North Pacific halibut | Certified | USA | Longlines | Passive gears | Large scale |
| MSC_Reports_38 | Pelagic Freezer Trawler Association North Sea herring | Certified | Europe | Pelagic trawls | Active gears | Large scale |
| MSC_Reports_39 | Pelagic Freezer Trawler Association North Sea herring | Certified | Europe | Pelagic trawls | Active gears | Large scale |
| MSC_Reports_40 | US North Pacific sablefish | Certified | USA | Longlines | Passive gears | Large scale |
| MSC_Reports_41 | Lake Hjälmaren pikeperch fish-trap and gillnet | Certified | Sweden | Pots & traps | Passive gears | Small scale |
| MSC_Reports_42 | Lake Hjälmaren pikeperch fish-trap and gillnet | Certified | Sweden | Pots & traps | Passive gears | Small scale |
| MSC_Reports_43 | Lake Hjälmaren pikeperch fish-trap and gillnet | Certified | Sweden | Pots & traps | Passive gears | Small scale |
| MSC_Reports_44 | Patagonian scallop (*Zygochlamys patagonica*) bottom otter trawl fishery | Certified | Argentina | Bottom trawls & dredges | Active gears | Large scale |
| MSC_Reports_45 | Patagonian scallop (*Zygochlamys patagonica*) bottom otter trawl fishery | Certified | Argentina | Bottom trawls & dredges | Active gears | Large scale |
| MSC_Reports_46 | AAFA and WFOA Pacific albacore tuna fisheries | Certified | USA | Other hooks & lines | Passive gears | Large scale |
| MSC_Reports_47 | AAFA and WFOA Pacific albacore tuna fisheries | Certified | USA | Other hooks & lines | Passive gears | Large scale |
| MSC_Reports_48 | AAFA and WFOA Pacific albacore tuna fisheries | Certified | USA | Other hooks & lines | Passive gears | Large scale |
| MSC_Reports_49 | North Eastern Inshore Fisheries and Conservation Authority sea bass | Withdrawn | UK | Entangling nets | Passive gears | Small scale |
| MSC_Reports_50 | North Eastern Inshore Fisheries and Conservation Authority sea bass | Withdrawn | UK | Entangling nets | Passive gears | Small scale |
| MSC_Reports_51 | Oregon and Washington pink shrimp | Certified | USA | Bottom trawls & dredges | Active gears | Large scale |
| MSC_Reports_52 | SPFPO Swedish North Sea herring | Certified | Sweden | Purse seines | Active gears | Large scale |
| MSC_Reports_53 | SPFPO Swedish North Sea herring | Certified | Sweden | Purse seines | Active gears | Large scale |
| MSC_Reports_54 | Lakes and Coorong, South Australia | Certified | Australia | Entangling nets | Passive gears | Small scale |
| MSC_Reports_55 | Lakes and Coorong, South Australia | Certified | Australia | Entangling nets | Passive gears | Small scale |
| MSC_Reports_56 | Norway North Sea Saithe; Norway North-East Arctic Saithe | Certified | Norway | Bottom trawls & dredges | Active gears | Large scale |
| MSC_Reports_57 | PFA & SPSG North Sea Herring | Certified | Europe | Pelagic trawls | Active gears | Large scale |
| MSC_Reports_58 | PFA & SPSG North Sea Herring | Certified | Europe | Pelagic trawls | Active gears | Large scale |
| MSC_Reports_59 | Gulf of St Lawrence northern shrimp trawl fishery Esquiman Channel | Certified | Canada | Bottom trawls & dredges | Active gears | Large scale |
| MSC_Reports_60 | Gulf of St Lawrence northern shrimp trawl fishery Esquiman Channel | Certified | Canada | Bottom trawls & dredges | Active gears | Large scale |
| MSC_Reports_61 | Kyoto Danish Seine Fishery Federation flathead flounder | Certified | Japan | Bottom trawls & dredges | Active gears | Large scale |
| MSC_Reports_62 | Kyoto Danish Seine Fishery Federation flathead flounder | Certified | Japan | Bottom trawls & dredges | Active gears | Large scale |
| MSC_Reports_63 | Gulf of St Lawrence northern shrimp trawl fishery Esquiman Channel | Certified | Canada | Bottom trawls & dredges | Active gears | Large scale |
| MSC_Reports_64 | Gulf of St Lawrence northern shrimp trawl fishery Esquiman Channel | Certified | Canada | Bottom trawls & dredges | Active gears | Large scale |
| MSC_Reports_65 | Germany North Sea saithe trawl | Certified | Germany | Bottom trawls & dredges | Active gears | Large scale |
| MSC_Reports_66 | Germany North Sea saithe trawl | Certified | Germany | Bottom trawls & dredges | Active gears | Large scale |
| MSC_Reports_67 | SPSG Ltd western component of north east Atlantic mackerel | Suspended | UK | Pelagic trawls | Active gears | Large scale |
| MSC_Reports_68 | SPSG Ltd western component of north east Atlantic mackerel | Suspended | UK | Pelagic trawls | Active gears | Large scale |
| MSC_Reports_69 | Domstein Longliner Partners North East Arctic fisheries | Withdrawn | Norway | Longlines | Passive gears | Large scale |
| MSC_Reports_70 | Domstein Longliner Partners North East Arctic fisheries | Withdrawn | Norway | Longlines | Passive gears | Large scale |
| MSC_Reports_71 | South Africa hake trawl | Certified | South Africa | Bottom trawls & dredges | Active gears | Large scale |
| MSC_Reports_72 | Hastings fleet Dover sole and Plaice | Certified | UK | Entangling nets | Passive gears | Small scale |
| MSC_Reports_73 | Hastings fleet Dover sole and Plaice | Certified | UK | Entangling nets | Passive gears | Small scale |
| MSC_Reports_74 | AAFA and WFOA Pacific albacore tuna fisheries | Certified | USA | Other hooks & lines | Passive gears | Large scale |
| MSC_Reports_75 | AAFA and WFOA Pacific albacore tuna fisheries | Certified | USA | Other hooks & lines | Passive gears | Large scale |
| MSC_Reports_76 | Mexico Baja California red rock lobster | Certified | Mexico | Pots & traps | Passive gears | Small scale |
| MSC_Reports_77 | Scottish Fisheries Sustainable Accreditation Group (SFSAG) North Sea haddock | Certified | UK | Bottom trawls & dredges | Active gears | Large scale |
| MSC_Reports_78 | Unidentified fishery | - | Undetermined | Pots & traps | Passive gears | Small scale |
| MSC_Reports_79 | Ekofish Group-North Sea twin rigged otter trawl plaice | Certified | Netherlands | Bottom trawls & dredges | Active gears | Large scale |
| MSC_Reports_80 | Oregon Dungeness crab | Withdrawn | USA | Pots & traps | Passive gears | Small scale |
| MSC_Reports_81 | PNA Western and Central Pacific skipjack and yellowfin, unassociated / non FAD set, tuna purse seine | Certified | Undetermined | Purse seines | Active gears | Large scale |
| MSC_Reports_82 | Mexico Baja California red rock lobster | Certified | Mexico | Pots & traps | Passive gears | Small scale |
| MSC_Reports_83 | Tosakatsuo Suisan skipjack tuna | Withdrawn | Japan | Other hooks & lines | Passive gears | Large scale |
| MSC_Reports_84 | Pescafria-Pesquera Rodriguez Barents Sea cod | Withdrawn | Spain | Bottom trawls & dredges | Active gears | Large scale |
| MSC_Reports_85 | Unidentified fishery | - | Undetermined | Entangling nets | Passive gears | Small scale |
| MSC_Reports_86 | DFPO Denmark Eastern Baltic cod | Suspended | Denmark | Undetermined | Undetermined | Large scale |
| MSC_Reports_87 | Small Pelagics Fishery in Sonora, Gulf of California | Certified | Mexico | Purse seines | Active gears | Large scale |
| MSC_Reports_88 | Suriname Atlantic seabob shrimp | Certified | Suriname | Bottom trawls & dredges | Active gears | Large scale |
| MSC_Reports_89 | Unidentified fishery | - | Undetermined | Entangling nets | Passive gears | Undetermined |
| MSC_Reports_90 | DFPO Denmark Eastern Baltic cod | Suspended | Denmark | Bottom trawls & dredges | Active gears | Large scale |
| MSC_Reports_91 | Normandy and Jersey lobster | Certified | Europe | Pots & traps | Passive gears | Small scale |
| MSC_Reports_92 | Alaska flatfish - Bering Sea and Aleutian Islands | Certified | USA | Bottom trawls & dredges | Active gears | Large scale |
| MSC_Reports_93 | Dutch rod and line fishery for sea bass | Withdrawn | Netherlands | Other hooks & lines | Passive gears | Small scale |
| MSC_Reports_94 | Oregon Dungeness crab | Withdrawn | USA | Pots & traps | Passive gears | Small scale |
| MSC_Reports_95 | Suriname Atlantic seabob shrimp | Certified | Suriname | Bottom trawls & dredges | Active gears | Large scale |
| MSC_Reports_96 | Osprey Trawlers North Sea twin-rigged plaice | Certified | Netherlands | Bottom trawls & dredges | Active gears | Large scale |
| MSC_Reports_97 | Scottish Fisheries Sustainable Accreditation Group (SFSAG) North Sea haddock | Certified | UK | Bottom trawls & dredges | Active gears | Large scale |
| MSC_Reports_98 | AAFA and WFOA Pacific albacore tuna fisheries | Certified | USA | Other hooks & lines | Passive gears | Large scale |
| MSC_Reports_99 | South Brittany sardine purse seine | Certified | France | Purse seines | Active gears | Large scale |
| MSC_Reports_100 | Oregon Dungeness crab | Withdrawn | USA | Pots & traps | Passive gears | Small scale |
| MSC_Reports_101 | Unidentified fishery | - | Undetermined | Undetermined (active) | Active gears | Large scale |
| MSC_Reports_102 | Mexico Baja California red rock lobster | Certified | Mexico | Pots & traps | Passive gears | Small scale |
| MSC_Reports_103 | Maldives pole & line tuna | Certified | Maldives | Other hooks & lines | Passive gears | Large scale |
| MSC_Reports_104 | Maldives pole & line tuna | Certified | Maldives | Other hooks & lines | Passive gears | Undetermined |
| MSC_Reports_105 | Unidentified fishery | - | Undetermined | Entangling nets | Passive gears | Small scale |
| MSC_Reports_106 | Vietnam Ben Tre clam hand gathered | Certified | Vietnam | Hand-operated gears | Passive gears | Small scale |
| MSC_Reports_107 | Maldives pole & line tuna | Certified | Maldives | Other hooks & lines | Passive gears | Large scale |
| MSC_Reports_108 | Razor Clam fishery from Ria de Pontevedra | Suspended | Spain | Hand-operated gears | Passive gears | Small scale |
| MSC_Reports_109 | Atlantic deep sea red crab | Withdrawn | USA | Pots & traps | Passive gears | Large scale |
| MSC_Reports_110 | Maine lobster trap | Certified | USA | Pots & traps | Passive gears | Small scale |
| MSC_Reports_111 | Maldives pole & line tuna | Certified | Maldives | Other hooks & lines | Passive gears | Undetermined |
| MSC_Reports_112 | Norway North East Arctic cod | Certified | Norway | Entangling nets | Passive gears | Undetermined |
| MSC_Reports_113 | Artisanal fishing nets on Mafia Island, Tanzania | Never certified | Tanzania | Entangling nets | Passive gears | Small scale |
| MSC_Reports_114 | Unidentified UK fishery | - | UK | Undetermined (passive) | Passive gears | Small scale |
| MSC_Reports_115 | Hastings fleet Dover sole and Plaice | Certified | UK | Entangling nets | Passive gears | Small scale |
| MSC_Reports_116 | Sian Ka'an and Banco Chinchorro Biosphere Reserves spiny lobster | Withdrawn | Mexico | Pots & traps | Passive gears | Small scale |
| MSC_Reports_117 | Dutch rod and line fishery for sea bass | Withdrawn | Netherlands | Other hooks & lines | Passive gears | Small scale |
| MSC_Reports_118 | Maine lobster trap | Certified | USA | Pots & traps | Passive gears | Small scale |
| MSC_Reports_119 | Küstenfischer Nord eG Heiligenhafen Germany Eastern Baltic cod | Withdrawn | Germany | Bottom trawls & dredges | Active gears | Large scale |
| MSC_Reports_120 | Gambian tonguesole fishery | Never certified | Gambia | Entangling nets | Passive gears | Small scale |
| MSC_Reports_121 | Indian oil sardine fisheries | Never certified | India | Entangling nets | Passive gears | Small scale |
| MSC_Reports_122 | Suriname Atlantic seabob shrimp | Certified | Suriname | Bottom trawls & dredges | Active gears | Large scale |
| MSC_Reports_123 | Vietnam Ben Tre clam hand gathered | Certified | Vietnam | Hand-operated gears | Passive gears | Small scale |
| MSC_Reports_124 | Madagascar octopus fisheries | Never certified | Madagascar | Hand-operated gears | Passive gears | Small scale |
| MSC_Reports_125 | Ashtamudi Estuary short-necked clam | Certified | India | Hand-operated gears | Passive gears | Small scale |
| MSC_Reports_126 | Gambian tonguesole fishery | Never certified | Gambia | Entangling nets | Passive gears | Small scale |
| MSC_Reports_127 | Vietnam Ben Tre clam hand gathered | Certified | Vietnam | Hand-operated gears | Passive gears | Small scale |
| MSC_Reports_128 | Madagascar octopus fisheries | Never certified | Madagascar | Hand-operated gears | Passive gears | Small scale |
| MSC_Reports_129 | Vietnam Ben Tre clam hand gathered | Certified | Vietnam | Hand-operated gears | Passive gears | Small scale |
| MSC_Reports_130 | Mexico Baja California red rock lobster | Certified | Mexico | Pots & traps | Passive gears | Small scale |
| MSC_Reports_131 | Juan Fernández Rock lobster | Certified | Chile | Pots & traps | Passive gears | Small scale |
| MSC_Reports_132 | South Africa hake trawl | Certified | South Africa | Bottom trawls & dredges | Active gears | Large scale |
| MSC_Reports_133 | South Africa hake trawl | Certified | South Africa | Bottom trawls & dredges | Active gears | Large scale |
| MSC_Reports_134 | Annette Islands Reserve salmon | Certified | USA | Undetermined (passive) | Passive gears | Small scale |
| MSC_Reports_135 | Fiji albacore tuna longline | Certified | Fiji | Longlines | Passive gears | Large scale |
| MSC_Reports_136 | Chilean mussel fishery and suspended culture Toralla S.A and Cultivos Toralla S.A | Certified | Chile | Others | Passive gears | Large scale |
| MSC_Reports_137 | Madagascar octopus fisheries | Never certified | Madagascar | Hand-operated gears | Passive gears | Small scale |
| MSC_Reports_138 | Vietnam Ben Tre clam hand gathered | Certified | Vietnam | Hand-operated gears | Passive gears | Small scale |
| MSC_Reports_139 | Normandy and Jersey lobster | Certified | Europe | Pots & traps | Passive gears | Small scale |
| MSC_Reports_140 | Mexico Baja California red rock lobster | Certified | Mexico | Pots & traps | Passive gears | Small scale |
| MSC_Reports_141 | Gambian tonguesole fishery | Never certified | Gambia | Entangling nets | Passive gears | Small scale |
| MSC_Reports_142 | New Zealand hoki | Certified | New Zealand | Bottom trawls & dredges | Active gears | Large scale |
| MSC_Reports_143 | Ekofish Group-North Sea twin rigged otter trawl plaice | Certified | Netherlands | Bottom trawls & dredges | Active gears | Large scale |
| MSC_Reports_144 | Poland Eastern Baltic cod | Suspended | Poland | Undetermined (active) | Active gears | Large scale |
| MSC_Reports_145 | Unidentified fishery | Never certified | Undetermined | Undetermined (passive) | Passive gears | Small scale |
| MSC_Reports_146 | Normandy and Jersey lobster | Certified | Europe | Pots & traps | Passive gears | Small scale |
| MSC_Reports_147 | Unidentified fishery | - | Undetermined | Undetermined (passive) | Passive gears | Small scale |
| MSC_Reports_148 | OHV Dutch Waddenzee and Oosterschelde Hand Raked cockle | Certified | Netherlands | Hand-operated gears | Passive gears | Small scale |
| MSC_Reports_149 | Ashtamudi Estuary short-necked clam | Certified | India | Hand-operated gears | Passive gears | Small scale |
| MSC_Reports_150 | Madagascar octopus fisheries | Never certified | Madagascar | Hand-operated gears | Passive gears | Small scale |
| MSC_Reports_151 | Suriname Atlantic seabob shrimp | Certified | Suriname | Bottom trawls & dredges | Active gears | Large scale |
| MSC_Reports_152 | Oregon Dungeness crab | Withdrawn | USA | Pots & traps | Passive gears | Small scale |
| MSC_Reports_153 | Vietnam Ben Tre clam hand gathered | Certified | Vietnam | Hand-operated gears | Passive gears | Small scale |
| MSC_Reports_154 | Maldives pole & line tuna | Certified | Maldives | Other hooks & lines | Passive gears | Undetermined |
| MSC_Reports_155 | PNA Western and Central Pacific skipjack and yellowfin, unassociated / non FAD set, tuna purse seine | Certified | Undetermined | Purse seines | Active gears | Large scale |
| MSC_Reports_156 | Juan Fernández Rock lobster | Certified | Chile | Pots & traps | Passive gears | Small scale |
| MSC_Reports_157 | Küstenfischer Nord eG Heiligenhafen Germany Eastern Baltic cod | Withdrawn | Germany | Bottom trawls & dredges | Active gears | Large scale |
| MSC_Reports_158 | Suriname Atlantic seabob shrimp | Certified | Suriname | Bottom trawls & dredges | Active gears | Large scale |
| MSC_Reports_159 | South Africa hake trawl | Certified | South Africa | Bottom trawls & dredges | Active gears | Large scale |
| MSC_Reports_160 | Vietnam Ben Tre clam hand gathered | Certified | Vietnam | Hand-operated gears | Passive gears | Small scale |
| MSC_Reports_161 | Normandy and Jersey lobster | Certified | Europe | Pots & traps | Passive gears | Small scale |
| MSC_Reports_162 | Germany Eastern Baltic cod | Certified | Germany | Undetermined (active) | Active gears | Undetermined |
| MSC_Reports_163 | CVO North Sea plaice and sole | Certified | Netherlands | Bottom trawls & dredges | Active gears | Large scale |
| MSC_Reports_164 | Vietnam Ben Tre clam hand gathered | Certified | Vietnam | Hand-operated gears | Passive gears | Small scale |
| MSC_Reports_165 | South Africa hake trawl | Certified | South Africa | Bottom trawls & dredges | Active gears | Large scale |
| MSC_Reports_166 | Unidentified fishery | - | Undetermined | Others | Passive gears | Small scale |
| MSC_Reports_167 | Mexico Baja California red rock lobster | Certified | Mexico | Pots & traps | Passive gears | Small scale |
| MSC_Reports_168 | Maldives pole & line tuna | Certified | Maldives | Other hooks & lines | Passive gears | Large scale |
| MSC_Reports_169 | Unidentified fishery | - | Undetermined | Undetermined (passive) | Passive gears | Small scale |
| MSC_Reports_170 | CVO North Sea plaice and sole | Certified | Netherlands | Bottom trawls & dredges | Active gears | Large scale |
| MSC_Reports_171 | CVO North Sea plaice and sole | Certified | Netherlands | Bottom trawls & dredges | Active gears | Large scale |
| MSC_Reports_172 | Spencer Gulf king prawn | Certified | Australia | Bottom trawls & dredges | Active gears | Large scale |
| MSC_Reports_173 | Maldives pole & line tuna | Certified | Maldives | Other hooks & lines | Passive gears | Undetermined |
| MSC_Reports_174 | ISF Iceland golden redfish, blue ling and tusk | Certified | Iceland | Undetermined (active) | Active gears | Large scale |
| MSC_Reports_175 | South Africa hake trawl | Certified | South Africa | Bottom trawls & dredges | Active gears | Large scale |
| MSC_Reports_176 | CVO North Sea plaice and sole | Certified | Netherlands | Bottom trawls & dredges | Active gears | Large scale |
| MSC_Reports_177 | Australian Western rock lobster | Certified | Australia | Pots & traps | Passive gears | Large scale |
| MSC_Reports_178 | Mexico Baja California red rock lobster | Certified | Mexico | Pots & traps | Passive gears | Small scale |
| MSC_Reports_179 | Alaska pollock - Gulf of Alaska | Certified | USA | Pelagic trawls | Active gears | Large scale |
| MSC_Reports_180 | AAFA and WFOA Pacific albacore tuna fisheries | Certified | USA | Other hooks & lines | Passive gears | Large scale |
| MSC_Reports_181 | Zhangzidao scallop | Certified | China | Bottom trawls & dredges | Active gears | Large scale |
| MSC_Reports_182 | Gaspésie lobster Trap fishery | Certified | Canada | Pots & traps | Passive gears | Small scale |
| MSC_Reports_183 | Küstenfischer Nord eG Heiligenhafen Germany Eastern Baltic cod | Withdrawn | Germany | Bottom trawls & dredges | Active gears | Large scale |
| MSC_Reports_184 | Australian Western rock lobster | Certified | Australia | Pots & traps | Passive gears | Large scale |
| MSC_Reports_185 | Australian Western rock lobster | Certified | Australia | Pots & traps | Passive gears | Large scale |
| MSC_Reports_186 | Gaspésie lobster trap fishery | Certified | Canada | Pots & traps | Passive gears | Small scale |
| MSC_Reports_187 | Gaspésie lobster trap fishery | Certified | Canada | Pots & traps | Passive gears | Small scale |
| MSC_Reports_188 | Juan Fernández rock lobster | Certified | Chile | Pots & traps | Passive gears | Small scale |
| MSC_Reports_189 | Kyoto Danish Seine Fishery Federation flathead flounder | Certified | Japan | Bottom trawls & dredges | Active gears | Large scale |
| MSC_Reports_190 | Scottish Fisheries Sustainable Accreditation Group (SFSAG) North Sea haddock | Certified | UK | Bottom trawls & dredges | Active gears | Large scale |
| MSC_Reports_191 | ISF Iceland golden redfish, blue ling and tusk | Certified | Iceland | Undetermined (active) | Active gears | Large scale |
| MSC_Reports_192 | West Greenland coldwater prawn | Certified | Greenland | Bottom trawls & dredges | Active gears | Large scale |
| MSC_Reports_193 | British Columbia sockeye salmon | Certified | Canada | Purse seines | Active gears | Large scale |
| MSC_Reports_194 | Alaska pollock - Gulf of Alaska | Certified | USA | Pelagic trawls | Active gears | Large scale |
| MSC_Reports_195 | Vietnam Ben Tre clam hand gathered | Certified | Vietnam | Hand-operated gears | Passive gears | Small scale |
| MSC_Reports_196 | Gambian tonguesole fishery | Never certified | Gambia | Entangling nets | Passive gears | Small scale |
| MSC_Reports_197 | Ashtamudi Estuary short-necked clam | Certified | India | Hand-operated gears | Passive gears | Small scale |
| MSC_Reports_198 | Zhangzidao scallop | Certified | China | Bottom trawls & dredges | Active gears | Large scale |
| MSC_Reports_199 | Unidentified fishery | - | Undetermined | Undetermined | Undetermined | Small scale |
| MSC_Reports_200 | Norway North East Arctic cod | Certified | Norway | Undetermined | Undetermined | Undetermined |
| MSC_Reports_201 | Norway North East Arctic cod | Certified | Norway | Longlines | Passive gears | Large scale |
| MSC_Reports_202 | South Africa hake trawl | Certified | South Africa | Bottom trawls & dredges | Active gears | Large scale |
| MSC_Reports_203 | Grupo Regal Spain hake longline | Suspended | Spain | Longlines | Passive gears | Large scale |
| MSC_Reports_204 | Normandy and Jersey lobster | Certified | Europe | Pots & traps | Passive gears | Small scale |
| MSC_Reports_205 | Unidentified fishery | Certified | Undetermined | Undetermined | Undetermined | Large scale |
| MSC_Reports_206 | British Columbia sockeye salmon | Certified | Canada | Purse seines | Active gears | Large scale |
| MSC_Reports_207 | Kyoto Danish Seine Fishery Federation flathead flounder | Certified | Japan | Bottom trawls & dredges | Active gears | Large scale |
| MSC_Reports_208 | Maldives pole & line tuna | Certified | Maldives | Other hooks & lines | Passive gears | Large scale |
| MSC_Reports_209 | Vietnam Ben Tre clam hand gathered | Certified | Vietnam | Hand-operated gears | Passive gears | Small scale |
| MSC_Reports_210 | Kenyan rock lobster fishery | Never certified | Kenya | Pots & traps | Passive gears | Small scale |
| MSC_Reports_211 | Unidentified fishery | Never certified | Undetermined | Entangling nets | Passive gears | Small scale |
| MSC_Reports_212 | Unidentified fishery | - | Undetermined | Undetermined | Undetermined | Undetermined |
| MSC_Reports_213 | Unidentified fishery | - | Undetermined | Entangling nets | Passive gears | Small scale |
| MSC_Reports_214 | Peel Harvey Estuarine Fishery: Recreational and Commercial blue swimmer crab and Commercial sea mull | Certified | Australia | Entangling nets | Passive gears | Small scale |
| MSC_Reports_215 | DFPO Denmark North Sea & Skagerrak cod & saithe | Certified | Denmark | Bottom trawls & dredges | Active gears | Large scale |
| MSC_Reports_216 | Ashtamudi Estuary short-necked clam | Certified | India | Hand-operated gears | Passive gears | Small scale |
| MSC_Reports_217 | ISF Iceland cod | Certified | Iceland | Longlines | Passive gears | Small scale |
| MSC_Reports_218 | Normandy and Jersey lobster | Certified | Europe | Pots & traps | Passive gears | Small scale |
| MSC_Reports_219 | Western Asturias octopus traps fishery of artisanal Cofradias | Certified | Spain | Pots & traps | Passive gears | Small scale |
| MSC_Reports_220 | MINSA North East Atlantic mackerel | Certified | UK, Denmark, Ireland, Norway, Netherlands, Germany, France, Lithuania, Sweden | Pelagic trawls | Active gears | Large scale |
| MSC_Reports_221 | PNA Western and Central Pacific skipjack and yellowfin, unassociated / non FAD set, tuna purse seine | Certified | Undetermined | Purse seines | Active gears | Large scale |
| MSC_Reports_222 | Walker Seafood Australian albacore, yellowfin tuna, and swordfish longline | Certified | Australia | Longlines | Passive gears | Large scale |
| MSC_Reports_223 | Maldives pole & line tuna | Certified | Maldives | Other hooks & lines | Passive gears | Large scale |
| MSC_Reports_224 | Madagascar octopus fisheries | Never certified | Madagascar | Hand-operated gears | Passive gears | Small scale |
| MSC_Reports_225 | Unidentified fishery | Never certified | Undetermined | Undetermined (passive) | Passive gears | Small scale |
| MSC_Reports_226 | FROM Nord North Sea and Eastern Channel trammel net sole | Certified | France | Entangling nets | Passive gears | Small scale |
| MSC_Reports_227 | Dongshan county multispecies fishery | Never certified | China | Undetermined | Undetermined | Undetermined |
| MSC_Reports_228 | Ashtamudi Estuary short-necked clam | Certified | India | Hand-operated gears | Passive gears | Small scale |
| MSC_Reports_229 | Unidentified Canadian fishery | - | Canada | Undetermined | Undetermined | Undetermined |
| MSC_Reports_230 | South Brittany sardine purse seine | Certified | France | Purse seines | Active gears | Large scale |
| MSC_Reports_231 | Alaska pollock - Bering Sea and Aleutian Islands | Certified | USA | Pelagic trawls | Active gears | Large scale |
| MSC_Reports_232 | Mexico Baja California red rock lobster | Certified | Mexico | Pots & traps | Passive gears | Small scale |
| MSC_Reports_233 | Kyoto Danish Seine Fishery Federation flathead flounder | Certified | Japan | Bottom trawls & dredges | Active gears | Large scale |
| MSC_Reports_234 | Vietnam Ben Tre clam hand gathered | Certified | Vietnam | Hand-operated gears | Passive gears | Small scale |
| MSC_Reports_235 | Suriname Atlantic seabob shrimp | Certified | Suriname | Bottom trawls & dredges | Active gears | Large scale |
| MSC_Reports_236 | Ashtamudi Estuary short-necked clam | Certified | India | Hand-operated gears | Passive gears | Small scale |
| MSC_Reports_237 | SPSG Ltd North Sea herring | Certified | UK | Pelagic trawls | Active gears | Large scale |
| MSC_Reports_238 | Ashtamudi Estuary short-necked clam | Certified | India | Hand-operated gears | Passive gears | Small scale |
| MSC_Reports_239 | ISF Iceland cod | Certified | Iceland | Longlines | Passive gears | Small scale |
| MSC_Reports_240 | Falkland Island toothfish | Certified | UK | Longlines | Passive gears | Large scale |
| MSC_Reports_241 | Vietnam Ben Tre clam hand gathered | Certified | Vietnam | Hand-operated gears | Passive gears | Small scale |
| MSC_Reports_242 | Vietnam Ben Tre clam hand gathered | Certified | Vietnam | Hand-operated gears | Passive gears | Small scale |
| MSC_Reports_243 | Unidentified fishery | - | Undetermined | Undetermined (active) | Active gears | Large scale |
| MSC_Reports_244 | Norway North East Arctic cod | Certified | Norway | Undetermined (passive) | Passive gears | Small scale |
| MSC_Reports_245 | Unidentified fishery | - | Undetermined | Purse seines | Active gears | Large scale |
| MSC_Reports_246 | Western Australia abalone fishery | Certified | Australia | Hand-operated gears | Passive gears | Small scale |
| MSC_Reports_247 | Ashtamudi Estuary short-necked clam | Certified | India | Hand-operated gears | Passive gears | Small scale |
| MSC_Reports_248 | Norway North East Arctic cod | Certified | Norway | Undetermined (passive) | Passive gears | Small scale |
| MSC_Reports_249 | Unidentified fishery | - | Undetermined | Other hooks & lines | Passive gears | Small scale |
| MSC_Reports_250 | Normandy and Jersey lobster | Certified | Europe | Pots & traps | Passive gears | Small scale |
| MSC_Reports_251 | Unidentified fishery | - | Undetermined | Undetermined (passive) | Passive gears | Small scale |
| MSC_Reports_252 | Western Asturias octopus traps fishery of artisanal Cofradias | Certified | Spain | Pots & traps | Passive gears | Small scale |
| MSC_Reports_253 | Maldives pole & line tuna | Certified | Maldives | Other hooks & lines | Passive gears | Undetermined |
| MSC_Reports_254 | US West Coast limited entry groundfish trawl | Certified | USA | Bottom trawls & dredges | Active gears | Large scale |
| MSC_Reports_255 | Unidentified fishery | - | Undetermined | Undetermined (passive) | Passive gears | Small scale |
| MSC_Reports_256 | Suriname Atlantic seabob shrimp | Certified | Suriname | Bottom trawls & dredges | Active gears | Large scale |
| MSC_Reports_257 | Unidentified seaweed farming | - | Undetermined | Hand-operated gears | Passive gears | Small scale |
| MSC_Reports_258 | SARPC toothfish | Certified | France | Longlines | Passive gears | Large scale |
| MSC_Reports_259 | Lake Hjälmaren pikeperch fish-trap and gillnet | Certified | Sweden | Pots & traps | Passive gears | Small scale |
| MSC_Reports_260 | Alaska salmon | Certified | USA | Purse seines | Active gears | Large scale |
| MSC_Reports_261 | ISF Iceland cod | Certified | Iceland | Other hooks & lines | Passive gears | Small scale |
| MSC_Reports_262 | Unidentified Gambian fishery | Never certified | Gambia | Undetermined (passive) | Passive gears | Small scale |
| MSC_Reports_263 | Normandy and Jersey lobster | Certified | Europe | Pots & traps | Passive gears | Small scale |
| MSC_Reports_264 | ISF Iceland Cod | Certified | Iceland | Longlines | Passive gears | Small scale |
| MSC_Reports_265 | Australian Western rock lobster | Certified | Australia | Pots & traps | Passive gears | Large scale |
| MSC_Reports_266 | Japanese Pole and Line skipjack and albacore tuna fishery | Certified | Japan | Other hooks & lines | Passive gears | Large scale |
| MSC_Reports_267 | Alaska pollock - Bering Sea and Aleutian Islands | Certified | USA | Pelagic trawls | Active gears | Large scale |
| MSC_Reports_268 | Alaska pollock - Bering Sea and Aleutian Islands | Certified | USA | Pelagic trawls | Active gears | Large scale |
| MSC_Reports_269 | South Brittany sardine purse seine | Certified | France | Purse seines | Active gears | Large scale |
| MSC_Reports_270 | Norway North East Arctic cod | Certified | Norway | Undetermined (passive) | Passive gears | Small scale |
| MSC_Reports_271 | Western Australia abalone fishery | Certified | Australia | Hand-operated gears | Passive gears | Small scale |
| MSC_Reports_272 | Peel Harvey estuarine fishery: recreational and commercial blue swimmer crab and commercial sea mull | Certified | Australia | Entangling nets | Passive gears | Small scale |
| MSC_Reports_273 | Unidentified Gambian fishery | Never certified | Gambia | Entangling nets | Passive gears | Small scale |
| MSC_Reports_274 | Suriname Atlantic seabob shrimp | Certified | Suriname | Bottom trawls & dredges | Active gears | Large scale |
| MSC_Reports_275 | Unidentified Spanish fishery | Never certified | Spain | Entangling nets | Passive gears | Small scale |
| MSC_Reports_276 | Shetland & Scottish mainland rope grown mussel enhanced fishery | Certified | UK | Others | Passive gears | Small scale |
| MSC_Reports_277 | Normandy and Jersey lobster | Certified | Europe | Pots & traps | Passive gears | Small scale |
| MSC_Reports_278 | Maldives pole & line tuna | Certified | Maldives | Other hooks & lines | Passive gears | Small scale |
| MSC_Reports_279 | Western Australia abalone fishery | Certified | Australia | Hand-operated gears | Passive gears | Small scale |
| MSC_Reports_280 | Scottish Fisheries Sustainable Accreditation Group (SFSAG) North Sea cod | Certified | UK | Bottom trawls & dredges | Active gears | Large scale |
| MSC_Reports_281 | Unidentified South African fishery | Never certified | South Africa | Undetermined (passive) | Passive gears | Small scale |
| MSC_Reports_282 | Madagascar octopus fisheries | Never certified | Madagascar | Hand-operated gears | Passive gears | Small scale |
| MSC_Reports_283 | Norway North East Arctic cod | Certified | Norway | Undetermined (passive) | Passive gears | Small scale |
| MSC_Reports_284 | Alaska salmon | Certified | USA | Entangling nets | Passive gears | Small scale |
| MSC_Reports_285 | Unidentified fishery | - | Undetermined | Undetermined | Undetermined | Large scale |
| MSC_Reports_286 | North Sea Brown Shrimp | Certified | Netherlands | Bottom trawls & dredges | Active gears | Large scale |
| MSC_Reports_287 | Ashtamudi Estuary short-necked clam | Certified | India | Hand-operated gears | Passive gears | Small scale |
| MSC_Reports_288 | Ashtamudi Estuary short-necked clam | Certified | India | Hand-operated gears | Passive gears | Small scale |
| MSC_Reports_289 | Ishihara Marine Products albacore and skipjack pole and line fishery | Certified | Japan | Longlines | Passive gears | Large scale |
| MSC_Reports_290 | North Sea brown shrimp | Certified | Netherlands | Bottom trawls & dredges | Active gears | Large scale |
| MSC_Reports_291 | Unidentified fishery | - | Undetermined | Undetermined (passive) | Passive gears | Small scale |
| MSC_Reports_292 | Unidentified fishery | - | Undetermined | Entangling nets | Passive gears | Small scale |
| MSC_Reports_293 | Falkland Island toothfish | Certified | UK | Longlines | Passive gears | Large scale |
| MSC_Reports_294 | Alaska salmon | Certified | USA | Purse seines | Active gears | Small scale |
| MSC_Reports_295 | Unidentified fishery | - | Undetermined | Undetermined (passive) | Passive gears | Small scale |
| MSC_Reports_296 | Indonesian pole and line tuna fishery | Never certified | Indonesia | Other hooks & lines | Passive gears | Small scale |
